# Design, Preparation, and Physicochemical Characterisation of Alginate-Based Honey-Loaded Topical Formulations

**DOI:** 10.3390/pharmaceutics15051483

**Published:** 2023-05-12

**Authors:** Md Lokman Hossain, Lee Yong Lim, Katherine Hammer, Dhanushka Hettiarachchi, Cornelia Locher

**Affiliations:** 1 Division of Pharmacy, School of Allied Health, University of Western Australia, Crawley, WA 6009, Australia; mdlokman.hossain@research.uwa.edu.au (M.L.H.); lee.lim@uwa.edu.au (L.Y.L.); dhanushka.hettiarachchi@outlook.com (D.H.); 2 School of Biomedical Sciences, University of Western Australia, Crawley, WA 6009, Australia; katherine.hammer@uwa.edu.au; 3 Cooperative Research Centre for Honey Bee Products Limited, 128 Yanchep Beach Road, Yanchep, WA 6035, Australia

**Keywords:** honey, honey-loaded formulation, physicochemical characteristics, High-Performance Thin-Layer Chromatography

## Abstract

Honey has widespread use as a nutritional supplement and flavouring agent. Its diverse bioactivities, including antioxidant, antimicrobial, antidiabetic, anti-inflammatory, and anticancer properties, have also made it an aspirant natural product for therapeutic applications. Honey is highly viscous and very sticky, and its acceptance as a medicinal product will require formulation into products that are not only effective but also convenient for consumers to use. This study presents the design, preparation, and physicochemical characterisation of three types of alginate-based topical formulations incorporating a honey. The honeys applied were from Western Australia, comprising a Jarrah honey, two types of Manuka honeys, and a Coastal Peppermint honey. A New Zealand Manuka honey served as comparator honey. The three formulations were a pre-gel solution consisting of 2–3% (*w*/*v*) sodium alginate solution with 70% (*w*/*v*) honey, as well as a wet sheet and a dry sheet. The latter two formulations were obtained by further processing the respective pre-gel solutions. Physical properties of the different honey-loaded pre-gel solutions (i.e., pH, colour profile, moisture content, spreadability, and viscosity), wet sheets (i.e., dimension, morphology, and tensile strength) and dry sheets (i.e., dimension, morphology, tensile strength, and swelling index) were determined. High-Performance Thin-Layer Chromatography was applied to analyse selected non-sugar honey constituents to assess the impacts of formulation on the honey chemical composition. This study demonstrates that, irrespective of the honey type utilised, the developed manufacturing techniques yielded topical formulations with high honey content while preserving the integrity of the honey constituents. A storage stability study was conducted on formulations containing the WA Jarrah or Manuka 2 honey. The samples, appropriately packaged and stored over 6 months at 5, 30, and 40 °C, were shown to retain all physical characteristics with no loss of integrity of the monitored honey constituents.

## 1. Introduction

Honey is produced by bees (*Apis mellifera*) from the nectar of flowers or honeydew and is without doubt one of the most appreciated natural food products. Honey is a complex mixture consisting of small amounts (in total approx. 3%) of numerous compounds distributed within a viscous matrix of supersaturated sugar (75–85% of the total solid) solution with a water content of between 13 and 21% [1,2]. The leading sugars in honey are fructose and glucose, with minor amounts of other mono-, di-, and oligosaccharides (e.g., maltose, sucrose, nigerose, isomaltose, turanose, and maltulose) [1,3,4]. The small fraction (about 3%) of non-sugar honey constituents comprises amino acids, minerals (e.g., Fe^2+^, Mg^2+^, Ca^2+^, Cu^+^, Mn^2+^, P^3+^, K^+^, Na^+^, and Zn^2+^), vitamins (e.g., vitamin B_6_, vitamin C, thiamine, niacin, and riboflavin), enzymes and other proteins, carotenoid-like substances, simple phenolic acids (e.g., gallic acid, ellagic acid, protocatechuic acid, syringic acid, benzoic acid, 4-hydroxybenzoic acid, chlorogenic acid, vanillic acid, caffeic acid, and *p*-coumaric acid), and polyphenolic compounds including flavonoids (e.g., quercetin, kaemferol, myricetin, rutin, apigenin, and luteolin) [1,2,3,4]. As well as being a food product, a sweetener, and flavouring agent, honey has also been applied throughout human history for medicinal purposes [1,2,3,4]. Honey has been demonstrated to exhibit a range of bioactivities, including antimicrobial, antioxidant, anti-inflammatory, anticancer, antidiabetic, antihyperlipidemic, antiulcer, as well as wound healing activities [5,6,7,8,9,10,11]. This study focuses on the incorporation of honey into formulations that could be used as wound dressings.

A wide range of wound dressings are commercially available [12]. Some of these dressings incorporate antimicrobial agents which may possess cytotoxic effects, especially after prolonged treatment, leading ultimately to a delay in wound healing [12,13]. Other dressings adhere to the wound surface that then damage the newly formed epithelium [12,13]. There is, therefore, scope for the development of alternative wound dressings, in particular formulations incorporating natural products such as honey, to overcome some of these limitations associated with conventional dressings [13]. Honey is a preferred choice as it has been widely used since ancient times to treat wounds [14]. There is also evidence that honey can contribute to the four stages of wound healing—haemostasis, inflammation, proliferation, and remodelling—and it has a positive impact on the natural physiology of wound healing by reducing oedema and wound exudation [15]. Furthermore, honey has been reported to promote collagen synthesis, angiogenesis, autolytic debridement, deodorizing of malodorous wounds, and growth of fibroblasts and epithelial cells in wounds while also preventing scar tissue and keloid formation [16]. The wound healing effects of honey are mainly related to its high osmolarity and acidity, its ability to generate hydrogen peroxide and nitric oxide upon contact with water, as well as the presence of so-called non-peroxide factors, which collectively exert antibacterial, anti-inflammatory, and antioxidant activities. Several studies have reported the successful application of honey to treat mild to moderate superficial and partial thickness burns [13,17,18,19,20], including a randomized clinical trial involving 105 patients that showed medical grade honey to confer significant clinical benefits in wound care [20]. It is, however, challenging to apply neat honey as a wound healing agent. Honey is viscous and inherently sticky, making it difficult to apply honey directly and uniformly on an open wound. Retention of honey at the application site to maintain therapeutic effect over an acceptable timeframe may also be difficult to achieve due to the liquefied nature of honey. To overcome these administrative issues, honey has been impregnated with other materials, such as collagen, gelatine, starch, cellulose, alginate, and agarose [21,22,23,24], to develop wound care products which, compared to neat honey, are more convenient to use and, therefore, more appealing to patients and health care professionals. The US Food and Drug Administration (FDA) has approved several honey-loaded wound care products formulated as hydrogels, dressings, ointments, and pastes [12]. There is, however, scope for more research into the development of honey-based wound care products. To date, the honeys in commercially available wound care products are largely Manuka honeys obtained from the plant genus *Leptospermum*. The incorporation of other honeys is less prevalent, even though honeys with phytochemical profiles different to Manuka honey may offer unique bioactivities for wound healing [12]. In particular, the honeys of Western Australia could be explored for potential application as wound care products. The state of Western Australia is home to eight of Australia’s fifteen biodiversity hotspots [25] and bees foraging on the state’s wide-ranging and, in parts, endemic floral species produce a range of unique bioactive honeys that possess antibacterial and antioxidant properties [25,26,27,28]. Aside from honey type, other limiting factors provide further impetus for developing improved honey-based wound care products. A significant number of commercial honey-based wound care products contain only low concentrations of honey while other products contain an admixture of honey and other natural agents that then makes it difficult to delineate claims of the efficacy of honey for wound management.

The present study aimed to design, prepare, and provide physicochemical characterisation of alginate-based formulations loaded with different bioactive honeys from Western Australia. Sodium alginate is a popular carrier for honey-based gels and wound dressings [29]. It is a natural product commercially available at low cost and is well suited for formulation into wound care products with relative ease and low cost of fabrication. Other advantages include alginate’s GRAS (Generally Regarded as Safe) status, high fluid absorptive capacity, low allergenicity, biocompatibility, and biodegradability [30], along with its capacity to form gels that can be crosslinked under mild conditions with divalent cations such as Ca^2+^ [31]. An advantage of crosslinking with Ca^2+^ is the potential to facilitate the in situ release of active honey constituents through an ion exchange process between Ca^2+^ in the gel and Na^+^ in the wound exudate [31,32]. Apart from this, alginate-based dressings also absorb a large amount of wound fluid, making them an excellent topical haemostatic agent [33].

A range of alginate-based wound dressings containing Western Australian honeys were prepared and evaluated in this study. The ability of alginate to form hydrogels that could be crosslinked with Ca^2+^ was exploited together with other processing techniques to prepare honey-loaded formulations of different textures that offer versatility in handling, transport, storage, and ease of use. Pre-gel solutions were prepared from simple mixtures of honey with aqueous sodium alginate solutions, whereas square sheets of soft gels (referred to as wet sheets) were produced by adding aqueous CaCl_2_ to the pre-gel solutions. Water was removed from the wet sheets by freeze drying to produce the corresponding firm dry sheets (referred to as dry sheets). The wet sheet is a composite hydrogel comprising a spongy network of hydrated alginate blocks crosslinked with Ca^2+^ and an interstitial aqueous solution of honey constituents, whereas the dry sheet is a xerogel of superior mechanical properties compared to the wet sheet. The physical properties of hydrogels can be improved through composite structures, which make them suitable for structural and high-performance applications [34,35]. Due to their key features such as softness, flexibility, biocompatibility, and high water content, composite gels are suitable for different biomedical applications [36,37,38,39,40].

The pH, colour profile, moisture content, spreadability, and viscosity of the honey-loaded pre-gel solutions, and the morphology, dimension and tensile strength of the wet and dry sheets, as well as the swelling index of dry sheets were evaluated immediately after manufacture and at specific time points over a 6-month storage period. This study also presents a novel approach by analysing selected honey constituents via High-Performance Thin-Layer Chromatography (HPTLC) to monitor the chemical compositional integrity of the honeys following their incorporation into the various medicinal formulations and upon storage of the formulations. The collective physicochemical data suggest that the pre-gel solutions, wet sheets, and dry sheets of the WA Jarrah honey and a WA Manuka honey 2 were stable for at least 6 months when stored at the different temperatures of 5, 30, and 40 °C.

## 2. Materials and Methods

### 2.1. Chemicals and Reagents

Chemicals and reagents, and their sources: 4,5,7-trihydroxyflavanone (Alfa Aesar, Lancaster, UK), anhydrous sodium sulphate (Merck KGaA, Darmstadt, Germany), low viscosity sodium alginate (BÜCHI Labortechnik AG, Meierseggstrasse 40, 9230 Flawil, Switzerland), and anhydrous magnesium sulphate (Scharlab S.L., Sentmenat, Barcelona, Spain). Hydroxyacetone (HA) (90%) and methylglyoxal (MGO) solution (40% *w*/*w* in water) were purchased from Sigma-Aldrich, Castle Hill, NSW, Australia. O-(2,3,4,5,6-Pentafluorobenzyl) hydroxylamine hydrochloride (PFBHA) (99%) was sourced from Alfa Aesar, Gymea, NSW, Australia.

Solvents and their sources: Methanol (Scharlau, Barcelona, Spain), dichloromethane (Merck KGaA, Darmstadt, Germany), acetonitrile (RCI Labscan, Bangkok, Thailand), toluene (APS Chemicals, Sydney, NSW, Australia), and ethyl acetate and formic acid (85%) (Ajax Finechem Pvt Ltd., Sydney, NSW, Australia). Sterile deionised water was used throughout to prepare the formulations.

### 2.2. Honey Samples

The honeys used in this study were two Western Australian (WA) Manuka (*Leptospermum* spp.) honeys: one WA Coastal Peppermint (*Agonis flexuosa*) honey and one WA Jarrah (*Eucalyptus marginata*) honey (Table 1). A New Zealand Manuka (*Leptospermum scoparium*) honey was included as a comparator honey given that most honey-based medicinal formulations currently on the market incorporate this honey (Table 1). The preliminary identification and documentation of the honey samples (e.g., date of harvest and floral sources) were carried out by the honey producer, mainly based on the availability of flowering nectar, the honeys’ organoleptic characteristics, and the location of the apiary/hives. After collection and assignment of a unique reference number, the samples were stored in plastic containers at room temperature and protected from light until investigation.

### 2.3. Preparation of Honey-Based Formulations

Three different types of alginate-based honey formulations were prepared for this study: (a) A pre-gel solution containing 70% *w*/*v* of honey in a sodium alginate solution; (b) A wet sheet obtained by crosslinking the respective pre-gel solutions with Ca^2+^; (c) A dry sheet prepared by freeze drying the respective wet sheet.

#### 2.3.1. Preparation of Pre-Gel Solutions

The honey-loaded pre-gel solutions contained 70% (*w*/*v*) of honey in 2 or 3% (*w*/*v*) sodium alginate solution. The formulations were prepared by dissolving 2 g (or 3 g in the case of Coastal Peppermint honey pre-gel formulations) of sodium alginate in 60 mL of sterile water in a 100 mL volumetric flask via magnetic stirring at 800 rpm for 30 min at room temperature. This was followed by the addition of 70 g of honey and water to a final volume of 100 mL. After thorough mixing for 3 h on the magnetic stirrer, the pre-gel solutions were stored at 5 °C in airtight sterile amber glass jars. The use of a more concentrated 3% sodium alginate solution for the Coastal Peppermint honey was prompted by the observed thinner consistency of its pre-gel solution compared to those of the other honeys when a 2% alginate solution was used.

#### 2.3.2. Preparation of Wet Sheets

Wet sheets were prepared by crosslinking the alginate in the pre-gel solutions with Ca^2+^. The fabrication process involved transferring 25 g of pre-gel solution into a square-shaped plastic container of 118.50 mm internal length, and adding 60 mL of aqueous CaCl_2_ solution (200 mM) to initiate crosslink formation. After 1 h of incubation at ambient temperature, a shape-retaining sponge-like hydrogel was obtained. Unreacted Ca^2+^ was removed by washing the hydrogel three times with 60 mL of sterile water. The resultant wet sheets were stored at 5 °C in airtight aluminium-based Mylar bags.

#### 2.3.3. Preparation of Dry Sheets

Dry sheets were prepared by freeze drying the wet sheets. To do this, the hydrogel immediately after manufacture and while still in the square plastic container was stored at −20 °C for 1 h followed by freeze drying over 24 h (Alpha 1-2 LDplus Freeze dryer, Martin Christ GmbH, Osterode am Harz, Germany). The freeze dryer was operated at −42 °C at a pressure of 0.1 mbar. The dry sheets were immediately transferred into airtight aluminium-based Mylar bags and stored at ambient temperature in a silica-gel-containing desiccator.

### 2.4. Physicochemical Evaluation of Honey and Honey-Based Formulations

The physicochemical properties and phytochemical composition of honeys are strongly influenced by their floral source and geographical origin. As they contribute to the honeys’ bioactivities, they constitute important characteristics that need to be determined for different honeys and by extension also the formulations prepared in this study that incorporate these honeys.

#### 2.4.1. Determination of pH

The pH of the neat honeys and the corresponding honey-loaded pre-gel solution was determined at room temperature using a calibrated pH meter (Eutech PC 2700-Eutech Instruments, Vernon Hills, IL, USA). Samples were prepared by adding 7.5 mL of carbon dioxide-free water to 1 g of honey or honey-loaded pre-gel solution and incubating the mixture at 37 °C for 15 min to aid dissolution [41]. pH measurement was conducted on three independent samples, and the results were expressed as mean ± SD.

#### 2.4.2. Colour Profile

Colour was determined by dissolving a honey or honey-loaded pre-gel solution in sterile distilled water to 50% (*w*/*v*) and measuring the optical density at 450 nm and 720 nm using a UV-Vis spectrophotometer (Cary 60, Agilent Technologies, Santa Clara, CA, USA) [42]. The difference in optical density between the two wavelengths was expressed in milli-absorbance units (mAU) to obtain the colour value. Colour values were determined in triplicates for all honeys and pre-gel solutions, both before and after the filtration of samples (0.7 μm syringe filter, Merck KGaA, Darmstadt, Germany), and the results were expressed as mean ± SD.

#### 2.4.3. Moisture Content

The moisture content of each honey and honey-loaded pre-gel solution was measured in triplicate using a refractometer (HI96801, Hanna Instruments, Smithfield, RI, USA) and expressed as a percentage (*w*/*w*) [41].

#### 2.4.4. Spreadability

**Figure 1:**
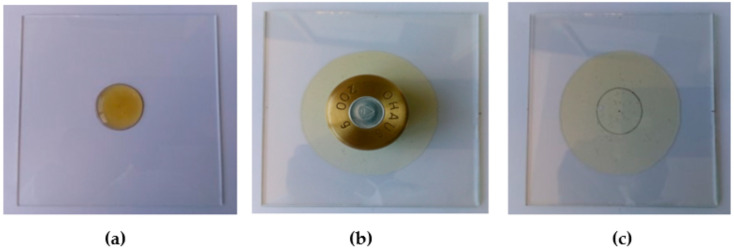
Determination of spreadability: (**a**) Placement of 1 g of honey sample within a circle of 2.4 cm diameter on a glass plate; (**b**) Placement of an upper glass plate with standardized weight on the sample; (**c**) Measurement of the final diameter of the sample after 5 min.

The spreadability of a honey and its pre-gel solution was determined following the method described by Chen et al. [43] with slight modifications. One gram of sample was placed within a circle of 2.4 cm diameter on a glass plate, and the spreading diameter was measured 5 min after placing on the sample another glass plate (mass 30.6 g) with a standardized weight of 200 g (Figure 1). Sample spreadability was calculated using the following formula:

S = m × l/t

where S is spreadability, m is the total mass (230.6 g) exerted on the sample, l is the diameter of the spreading sample (cm), and t is the time (5 min) at which the measurement was taken. The analysis was performed on three independent samples, and the results were expressed as mean ± SD.

#### 2.4.5. Rheology

The viscosity of a honey and its pre-gel solution was determined using a Modular Compact Rheometer (MCR) (Anton Paar-MCR 72, Graz, Austria) at 25 and 37 °C using a constant shear rate of 100 s^−1^. Analysis was carried out on triplicate samples and the results were expressed as mean ± SD.

#### 2.4.6. Physical Dimensions

**Figure 2:**
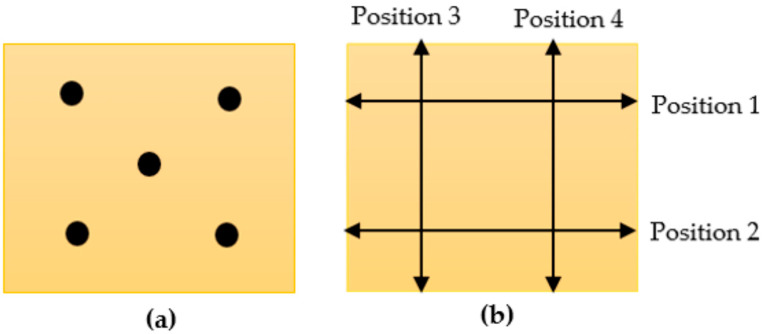
Determination of the dimensions of wet and dry sheets: (**a**) Positions for measuring thickness; (**b**) Positions for measuring length.

The thickness and length of the squarish honey-loaded wet and dry sheets were determined in triplicates using a digital vernier caliper (150 mm Vernier Caliper, Kincrome, Scoresby, VIC, Australia). For each sheet, thickness was determined as the mean of thickness measured at five different locations (Figure 2a), whereas the length was determined as the mean of length measurements taken at four positions (Figure 2b).

#### 2.4.7. Morphology

The gross morphology of the WA Jarrah honey-loaded wet and dry sheets was observed under a microscope (VEVOR^®^ stereo microscope, Rancho Cucamonga, CA, USA). The wet and dry sheets were placed directly onto the viewing platform of the microscope and images of the surface and cross-section of the sheets were taken at 5× magnification.

#### 2.4.8. Tensile Strength

The tensile strength of the honey-loaded wet and dry sheets was determined using a tensile tester UniVert (CellScale, Waterloo, ON, Canada) equipped with a 200 N load cell following the method described by Hervy et al. [44] with slight modifications. Test samples measuring 40 mm by 5 mm were cut from each sheet, and the force required to trigger the failure of each sample was recorded. Analysis was performed on triplicate samples and the data were expressed as mean ± SD.

#### 2.4.9. Swelling Index

The swelling index of the honey-loaded dry sheets was determined using the method described by El-Kased et al. [13] with minor modifications. Each dry sheet was weighed (W_O_) before it was immersed in 5 mL of sterile water in a glass container. At the time points of 10, 20, and 30 min, the sample was removed, gently blotted with kimtech wipes to remove excess water, and its weight was recorded (W_S_). At the sampling time points of 10 and 20 min, the sample after weighing was returned to the container for continued immersion in the liquid until the next sampling time point. At 30 min, the sample after weighing was dried at 40 °C (Memmert oven, GmbH + Co. KG, Büchenbach, Germany) to constant weight. The following equation was used to calculate the swelling index:

Swelling Index (%) = {(Ws − Wo)/Wo} × 100

where W_S_ was the weight of the swollen formulation at time t and W_O_ was its initial dry weight.

#### 2.4.10. Determination of Methylglyoxal (MGO) Content

Methylglyoxal (MGO), a major contributor to the antibacterial activity of *Leptospermum* spp. honey was quantified for the formulations loaded with the WA Manuka honeys and New Zealand Manuka honey. MGO was quantified using a validated HPLC assay described by Hossain et al. [45]. Studies have shown that high levels of MGO in Manuka honey stem from the presence of dihydroxyacetone (DHA) [46,47,48], identified as a direct precursor for MGO formation. DHA is produced by the plant or by microbes present in the flower [49], and dehydrates to MGO during honey maturation [47].

Briefly, a concentration of 0.5 g/mL solution of neat honey or corresponding honey-loaded formulation was prepared in deionised water. A total of 250 µL of the prepared sample solution was taken in a test tube followed by the addition of HA (250 µL). The resulting solution was thoroughly mixed using a vortex mixer (MX-S, DLAB Scientific Co., Ltd., Beijing, China), and allowed to stand for 1 h. Then, 1500 µL of PFBHA derivatising solution was added and mixed completely. Finally, 6 mL acetonitrile was added followed by the addition of deionised water to make the ultimate volume of 10 mL. The entire solution was properly mixed and then 1 mL of aliquot was taken for HPLC analysis.

### 2.5. Component Analysis

Component analysis was focused on the quantification of selected non-sugar constituents in each honey and corresponding formulations as the non-sugar constituents (e.g., phenolic, flavonoids, vitamins, and minerals) play a major role in the bioactivity of honey [12]. Component analysis was determined using High-Performance Thin-Layer Chromatography (HPTLC), which allows for the monitoring of selected bands representing individual honey constituents [50]. Briefly, 1 g of honey or honey-loaded formulation was dissolved in 2 mL of phosphate buffer solution and the resulting solution was extracted three times with 5 mL of a mixture of dichloromethane and acetonitrile (50:50 *v*/*v*). After the addition of MgCl_2_ anhydrous to the combined organic extracts followed by filtration, the extraction solvent was evaporated using compressed air and the resulting organic extract was reconstituted in 100 µL of methanol prior to HPTLC analysis. The chromatographic separation was performed on silica gel 60 F_254_ HPTLC glass plates in an automated development chamber (ADC2, CAMAG, Muttenz, Switzerland) using a mixture of toluene-ethyl acetate-formic acid, 1:6:1 (*v*/*v*) as the mobile phase. The obtained chromatographic results were documented using an HPTLC imaging device (TLC Visualizer, CAMAG) under white light, 254 nm and 366 nm, respectively. The chromatographic images were digitally processed and analysed using a specialized HPTLC software (visionCATS, CAMAG). The peak area of selected bands obtained from each honey-based formulation was compared with that of the corresponding bands in the respective neat honey and reported as a percentage. The analysis was carried out in triplicate for each formulation and the results were expressed as mean ± SD.

### 2.6. Stability Study

From the five types of honey used for the preparation of honey-loaded formulations, two representative honeys (Jarrah and WA Manuka 2 honey) and their corresponding formulations were subjected to a 6-month stability study. The samples were stored at three different temperatures (5, 30, and 40 °C) using capped amber glass tubes for the storage of neat honeys and pre-gel solutions, and aluminium-based Mylar bags (23 × 32 cm; Protection Experts Australia, Sydney, NSW, Australia) for the storage of the wet and dry sheets. Samples were removed monthly for the evaluation of physicochemical characteristics (pH, moisture content, spreadability, dimension, tensile strength, swelling index, MGO content, and component analysis by HPTLC). In addition, 5-hydroxymethylfurfural (HMF), an organic compound formed in honey upon processing and storage, in particular at elevated temperatures, was also determined using the HPTLC-based method described by Islam et al. [51]. HMF content in honey is widely recognised as a measure of honey freshness because HMF is typically present in negligible amounts in freshly harvested honeys whereas its concentration in honey tends to rise following processing and/or aging [52,53,54].

### 2.7. Data Analysis

Experiments were performed in triplicates, and the results were evaluated by a one-way or two-way analysis of variance (ANOVA) followed by Tukey’s honestly significant difference (TukeyHSD) test using GraphPad Prism 9.4.1 (GraphPad Software, San Diego, CA, USA). A *p*-value of less than 0.05 was considered statistically significant.

## 3. Results

### 3.1. Physicochemical Characterisation

#### 3.1.1. pH

The five honeys used in this study had similar pH values in a narrow acidic range of 4.56 to 4.75. Pre-gel solutions prepared by mixing the honeys with alginate solution (pH 6.90) also showed comparable pH values that were on average 0.76 units higher than the pH of the corresponding honeys (*p* < 0.0001) (Table 2).

#### 3.1.2. Colour Profile

Colour profiling (Table 2) showed the NZ Manuka honey to be the darkest in colour, followed by the two WA Manuka honeys, Jarrah honey, and Coastal Peppermint honey. The pre-gel solution formulations were 20–40% less intense in coloration than the corresponding honeys, likely due to the diluting effect of the alginate solution. When the pre-gel formulations were filtered, their coloration was up to 50% less than the respective neat honeys, suggesting that some coloured constituents might have been removed by filtration.

#### 3.1.3. Moisture Content

The neat honeys used in this study contained different moisture contents (*p* < 0.0001) that ranged from 17.30 to 20.30%. The pre-gel solutions had moisture content that was at least 2.5-fold higher than the corresponding honeys; however, there was no significant difference (*p* = 0.067) in moisture content among the pre-gel solutions containing the different honeys (Table 3).

#### 3.1.4. Spreadability

The spreadability of the five honeys was different (*p* < 0.0001) from one another, ranging in values from 268 to 368 g.cm/sec. The pre-gel solutions were 1.15 to 1.59 times more spreadable compared to the corresponding honeys; however, there was no difference in spreadability among the different honey-loaded pre-gel solutions (*p* = 0.025) (Table 4).

#### 3.1.5. Rheology

The neat honeys showed different (*p* < 0.0001) shearing behaviours to each other, and their rheological behaviour at 25 °C also differed from that at 37 °C (Table 5). As was expected, all the honeys became runnier at the higher temperature of 37 °C and their viscosity decreased. The pre-gel solutions also showed lower viscosity (*p* < 0.0001) at 37 °C compared to 25 °C. However, in contrast to the neat honeys, the pre-gel solutions had comparable viscosity irrespective of the type of honey loaded, and this was observed both at 25 °C (*p* = 0.075) and 37 °C (*p* = 0.369) (Table 5).

#### 3.1.6. Thickness and Length

The wet sheets had comparable thickness regardless of the type of honey loaded (*p* = 0.329); the mean thickness was about 2.0 mm for all the sheets (Table 6). Transformation of the wet sheet to dry sheet significantly reduced (*p* < 0.0001) the thickness to about 1.4 mm, and this was observed for all types of honey load (Table 6). The wet sheet also showed shrinkage of length by about 1 mm when freeze dried to give the dry sheet (*p* < 0.0001). However, similar to thickness, the length of the wet sheets as well as that of the dry sheets were no different (*p* = 0.081) across the different honey-loaded formulations (Table 6).

#### 3.1.7. Morphology

**Figure 3:**
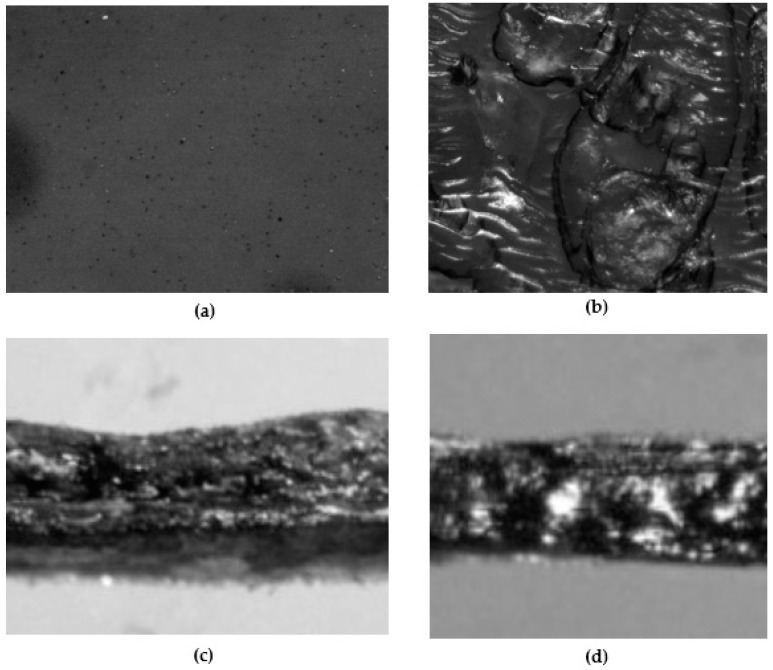
Gross morphology of the WA Jarrah honey-loaded wet and dry sheets as seen under a stereomicroscope (5× magnification): (**a**) Wet sheet (surface view); (**b**) Dry sheet (surface view); (**c**) Wet sheet (cross-sectional view); (**d**) Dry sheet (cross-sectional view).

The stereoscopic images of the surface and cross-section of the wet and dry sheets loaded with the WA Jarrah honey are shown in Figure 3. The wet sheet showed a homogenously uneven surface (Figure 3a) and edge (Figure 3c) with no remarkable features while the dry sheet showed a rough surface with raised nodules (Figure 3b). The thicknesses of the two sheets as seen under the microscope (Figure 3c,d) were comparable to the respective sheet thicknesses measured using the vernier caliper.

#### 3.1.8. Tensile Strength

**Figure 4:**
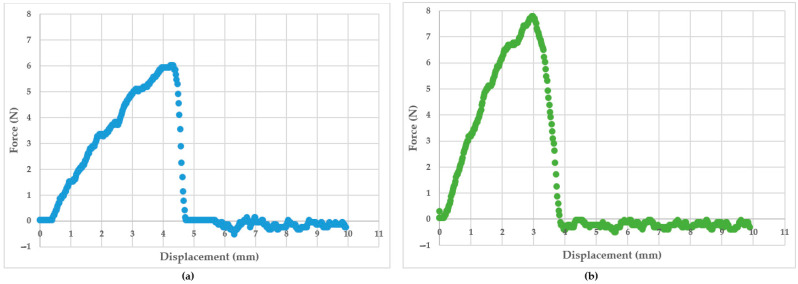
Representative force (N) and displacement (mm) curve: (**a**) honey-based wet sheet; (**b**) honey-based dry sheet.

**Figure 5:**
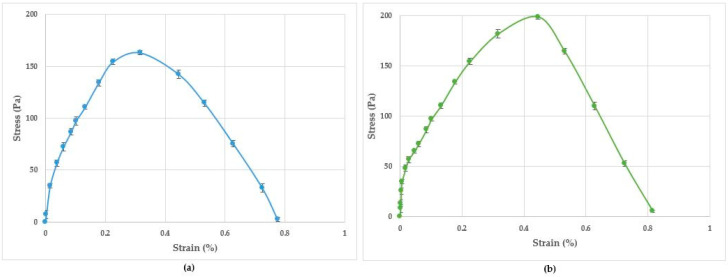
Representative tensile stress–strain curve: (**a**) honey-based wet sheet; (**b**) honey-based dry sheet.

Representative load–displacement curves for the wet and dry sheets are shown in Figure 4. The initial linear part of the load–displacement curve corresponds to the strain potential energy stored in the sample when a load was applied. When the applied load was high enough to create a new surface area, the introduced crack started to propagate until the test specimen failed catastrophically. Figure 5 shows the representative stress–strain curves for the wet and dry sheets and the recorded tensile strength is presented in Table 7. The tensile strength ranged from 100.11 to 111.15 Pa for the wet sheets and 185.28 to 200.45 Pa for the dry sheets. Irrespective of the type of honey load, the dry sheets consistently demonstrated tensile strength that was between 75 and 85% higher than that for the corresponding wet sheets.

#### 3.1.9. Swelling Index

Two variables, the honey type and incubation time, were taken into consideration to determine the swelling behaviour of the dry sheets. The degree of swelling represents the extent of water uptake by the sheets. All the dry sheet formulations had the ability to swell, suggesting that they were all able to absorb water when immersed in an aqueous environment. The swelling index of the dry sheets increased (*p* < 0.0001) from 10 to 20 min; however, no difference in the swelling index (*p* = 0.949) was observed from 20 to 30 min (Table 8), suggesting that the dry sheets had reached their maximum swelling capacity after 20 min incubation in the aqueous medium. To confirm this, the immersion of the dry sheet in the liquid medium was further prolonged to 45 min, and the swelling index measured at 45 min was no different to that measured at 30 min (Table 8).

#### 3.1.10. MGO Content

The MGO content varied across the three Manuka honeys (*p* < 0.0001) and ranged between 95 to 350 mg/kg (Table 9). However, the content of MGO in the three types of formulations (pre-gel solution, wet sheet, and dry sheet) was comparable (*p* > 0.05) to the amount found in the corresponding neat honey, indicating that the formulation process had no negative impact on MGO content.

### 3.2. Component Analysis

**Figure 6:**
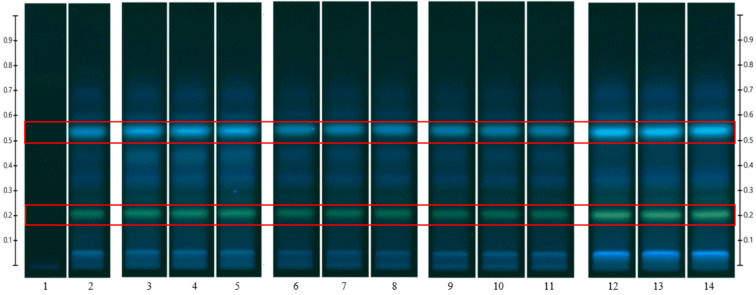
Jarrah (JAR) honey—red box indicates the monitored bands at Rf 0.20 and 0.53; Track 1—4,5,7-trihydroxyflavone (internal standard), Track 2—neat JAR honey extract (system suitability test), Tracks 3–5—neat JAR honey extracts, Tracks 6–8—JAR honey pre-gel solution extract, Tracks 9–11—JAR honey wet sheet extracts, and Tracks 12–14—JAR honey dry sheet extracts; image taken at 366 nm.

**Figure 7:**
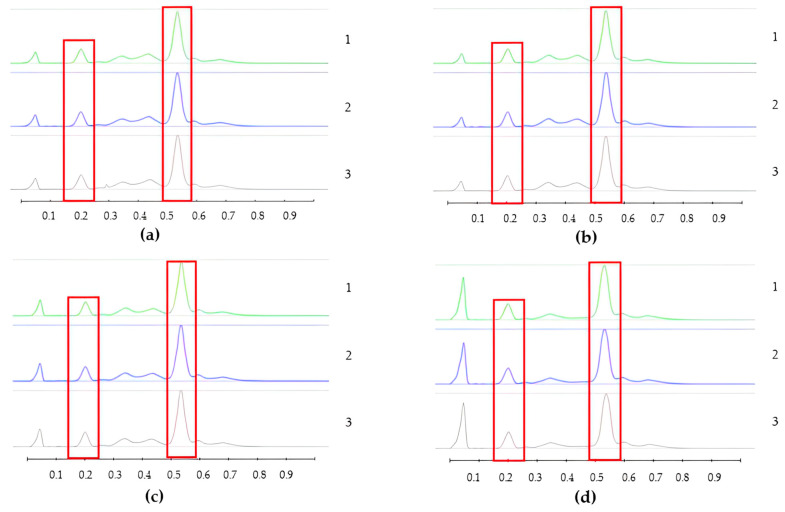
Peak profile: (**a**) Neat JAR honey extract; (**b**) JAR honey pre-gel solution extract; (**c**) JAR honey wet sheet extract; (**d**) JAR honey dry sheet extract. Red boxes highlight monitored bands (Rf Rf 0.20 and 0.53).

**Figure 8:**
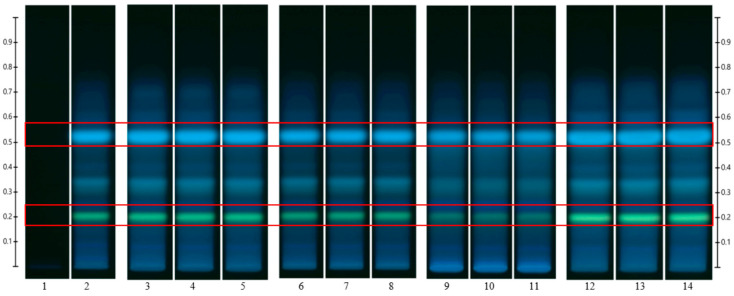
Coastal Peppermint (CP) honey—red box indicates the monitored bands at Rf 0.20 and 0.53; Track 1—4,5,7-trihydroxyflavone (internal standard), Track 2—neat CP honey extract (system suitability test), Tracks 3–5—neat CP honey extract, Tracks 6–8—CP honey pre-gel solution extract, Tracks 9–11—CP honey wet sheet extract, and Tracks 12–14—CP honey dry sheet extract; image taken at 366 nm.

**Figure 9:**
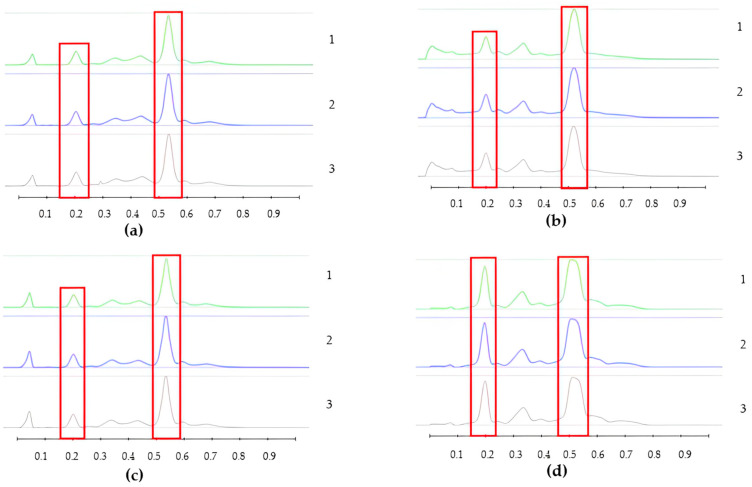
Peak profile: (**a**) Neat CP honey extract; (**b**) CP honey pre-gel solution extract; (**c**) CP honey wet sheet extract; (**d**) CP honey dry sheet extract. Red boxes highlight monitored bands (Rf 0.20 and 0.53).

**Figure 10:**
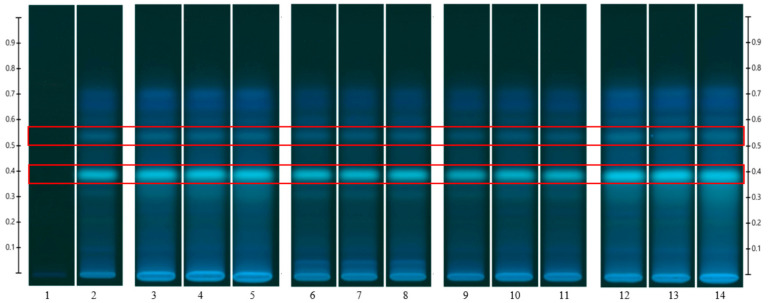
WA Manuka honey 1 (WAM1)—red box indicates the monitored bands at Rf 0.38 and 0.53; Track 1—4,5,7-trihydroxyflavone (internal standard), Track 2—WAM1 honey extract (system suitability test), Tracks 3–5—WAM1 honey extract, Tracks 6–8—WAM1 honey pre-gel solution extract, Tracks 9–11—WAM1 honey wet sheet extract, and Tracks 12–14—WAM1 honey dry sheet extract; image taken at 366 nm.

**Figure 11:**
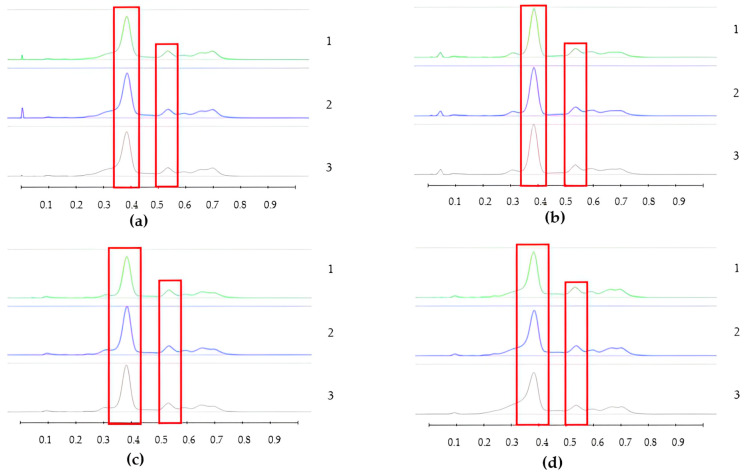
Peak profile: (**a**) Neat WAM1 honey extract; (**b**) WAM1 honey pre-gel solution extract; (**c**) WAM1 honey wet sheet extract; (**d**) WAM1 honey dry sheet extract. Red boxes highlight monitored bands (Rf 0.38 and 0.53).

**Figure 12:**
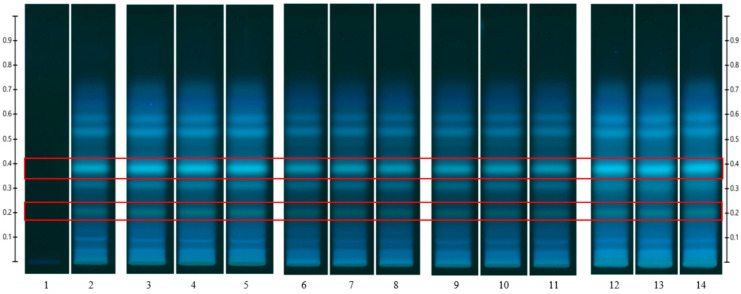
WA Manuka honey 2 (WAM2)—red box indicates the monitored bands at Rf 0.20 and 0.38; Track 1—4,5,7-trihydroxyflavone (internal standard), Track 2—WAM2 honey extract (system suitability test), Tracks 3–5—WAM2 honey extract, Tracks 6–8—WAM2 honey pre-gel solution extract, Tracks 9–11—WAM2 honey wet sheet extract, and Tracks 12–14—WAM2 honey dry sheet extract; image taken at 366 nm.

**Figure 13:**
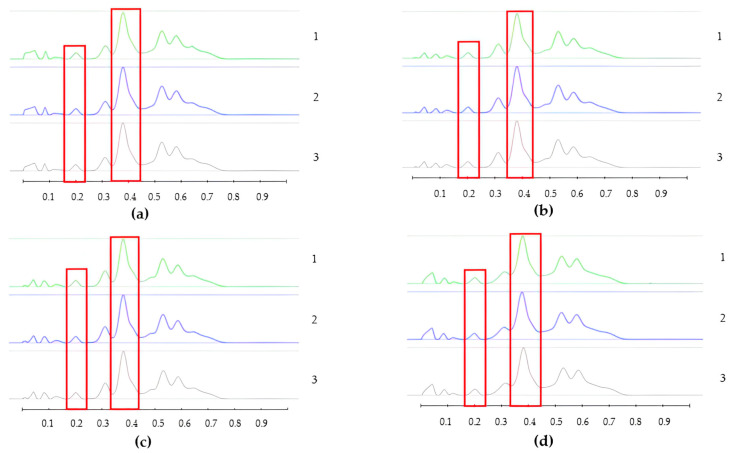
Peak profile: (**a**) Neat WAM2 honey extract; (**b**) WAM2 honey pre-gel solution extract; (**c**) WAM2 honey wet sheet extract; (**d**) WAM2 honey dry sheet extract. Red boxes highlight monitored bands (Rf 0.20 and 0.38).

**Figure 14:**
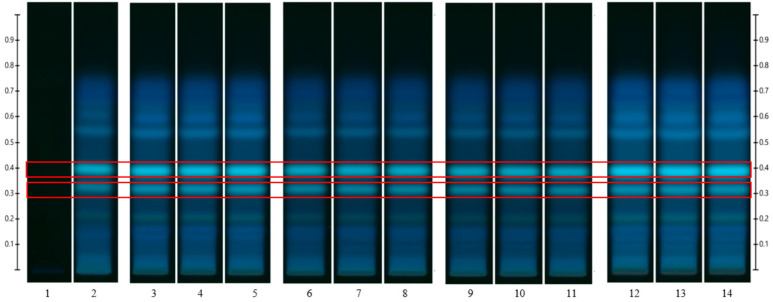
NZ Manuka (NZM) honey—red box indicates the monitored bands at Rf 0.32 and 0.39; Track 1—4,5,7-trihydroxyflavone (internal standard), Track 2—NZM honey extract (system suitability test), Tracks 3–5—NZM honey extract, Tracks 6–8—NZM honey pre-gel solution extract, Tracks 9–11—NZM honey wet sheet extract, and Tracks 12–14—NZM honey dry sheet extract; image taken at 366 nm.

**Figure 15:**
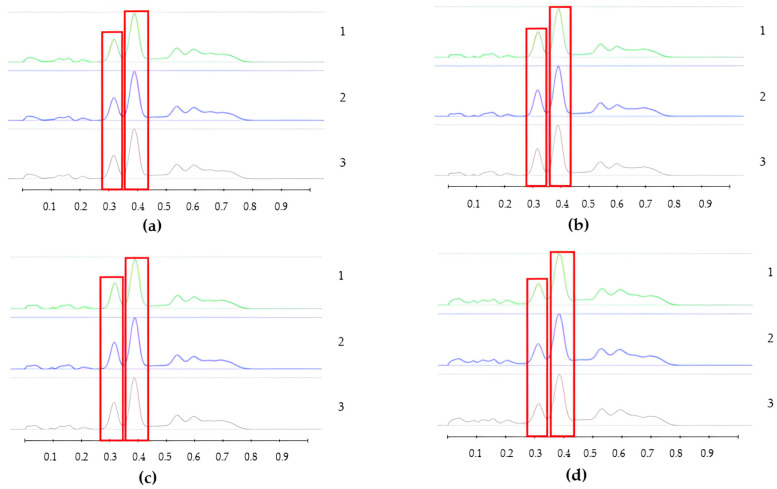
Peak profile: (**a**) Neat NZM honey; (**b**) NZM honey pre-gel solution formulation; (**c**) NZM honey wet sheet; (**d**) NZM honey dry sheet. Red boxes highlight monitored bands (Rf 0.32 and 0.39).

To analyse the potential impact of the formulation manufacturing process on the honeys’ chemical composition, selective non-sugar honey constituents characterised by specific HPTLC bands were monitored for each honey type, and their presence and concentration in each formulation extract was compared with the corresponding bands in the respective neat honey extract (Figure 6, Figure 7, Figure 8, Figure 9, Figure 10, Figure 11, Figure 12, Figure 13, Figure 14 and Figure 15). The selected bands for the five honeys and their respective formulations: (a) WA Jarrah honey at Rf 0.20 and 0.53; (b) Coastal Peppermint honey at Rf 0.20 and 0.53; (c) WA Manuka honey 1 at Rf 0.38 and 0.53; (d) WA Manuka honey 2 at Rf 0.20 and 0.38; (e) NZ Manuka honey at Rf 0.32 and 0.39 (Figure 6, Figure 8, Figure 10, Figure 12, and Figure 14, respectively). The peak area (AU) generated for each band in the respective honey extract was determined from the sample’s peak profile (Figure 7, Figure 9, Figure 11, Figure 13 and Figure 15) and compared to those obtained from the honey-based formulation extracts.

As can be seen from the data presented in Table 10, the monitored components in the formulations remained at more than 97% compared to neat honey, indicating that the manufacturing process did not lead to a significant decrease in their concentration in the formulation. The normalised peak areas appear to suggest that the monitored components in the dry sheets were present in much higher amounts (Table 10). However, it should be noted that the water content had been removed in the dry sheet by freeze drying. Indeed, when considering the respective areas under the curve of selected components in the wet and dry sheets on a per sheet basis rather than per g of formulation (Table 10), almost identical figures were obtained for the dry and wet sheets, indicating that the transformation of wet sheets into dry sheets by freeze drying did not lead to a loss in the monitored honey components.

### 3.3. Storage Stability

The 6-month stability data based on the physicochemical characterization of Jarrah and WA Manuka honey 2 and their corresponding honey-based formulations are presented in the supplementary file (Supplementary Materials Tables S1–S9). The pH, moisture content, and spreadability of samples stored at 6 months were comparable to their baseline data (Table 11). The thickness and length were not impacted by the storage conditions or the duration of storage. For example, for the wet sheets containing WA Jarrah honey, the thickness did not change at different storage temperatures (5, 30, and 40 °C; *p* = 0.884) and duration (baseline and 6 months; *p* = 0.310) (Table 12). The tensile strength of the wet and dry sheets was also found to be similar to their baseline values over the six months of storage (Table 12 and Table S6). Moreover, the swelling index of the dry sheets was unchanged at all the storage temperatures and durations (Table 13, Tables S7–S9).

The peak areas (baseline and 6 months) of the monitored bands are presented in Table 14. Moreover, for easier comparison, the peak areas of these selected bands were calculated per wet and per dry sheet. The HPTLC fingerprints of samples from the stability study are included as a supplementary file (Supplementary Materials Figures S1–S8). The monitored bands remained unchanged over the six-month storage period.

The MGO content of neat WA Manuka honey 2 and its corresponding formulations is presented in Table 15. The MGO content did not change in the neat honey nor in the formulations when stored at 5 °C; however, as expected, upon storage at 30 and 40 °C, a decrease of between 1 and 8% of MGO content could be noted. For example, the MGO content of neat WA Manuka honey 2 remained unchanged (*p* = 0.757) at 1 and 6 months of storage at 5 °C, whereas progressive declines in MGO content were observed for samples stored for increasing durations at 30 °C and 40 °C. Comparisons of samples stored for 1 month and 6 months at different temperatures indicate significant decreases in MGO content between samples stored at 5 °C and 30 °C (*p* < 0.0001) and also between samples stored at 30 °C and 40 °C (*p* < 0.0001), suggesting the degradation of MGO upon storage at 30 °C and 40 °C.

**Figure 16:**
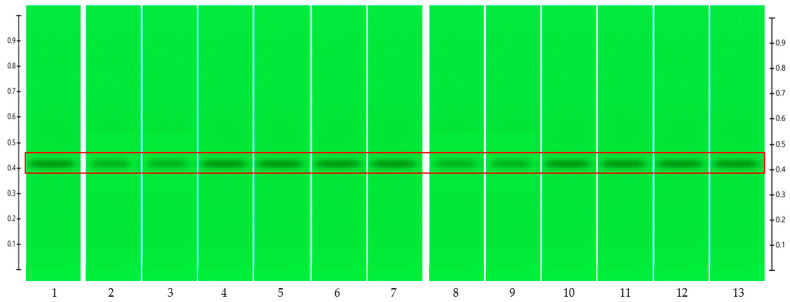
Representative HPTLC image taken at 254 nm after development with ethyl acetate. Track 1: HMF (1 mg/mL) aqueous solution; Track 2–7: WA Manuka honey 2 extracts at 1, 2, 3, 4, 5, and 6 months of storage at 30 °C; and Tracks 8–13: WA Manuka honey 2 at 1, 2, 3, 4, 5, and 6 months of storage at 40 °C. Red box highlights monitored band (Rf 0.41).

HMF was quantified using a HPTLC assay and the absorbance spectrum of a standard HMF solution and the corresponding band in a honey extract are shown in the Supplementary Materials Figure S9. HMF was not detected in the WA Jarrah and Manuka 2 honeys or the honey-loaded formulations immediately after manufacture or when stored at 5 °C for up to 6 months. HMF was, however, found in the WA Jarrah and Manuka 2 honeys and their corresponding formulations when stored for 1 month and longer at 30 °C and 40 °C (Figure 16).

## 4. Discussion

Due to its wide-ranging bioactivity profile, honey holds promise as a key natural ingredient in topical medicinal formulations for wound healing purposes [15,23,55,56]. The stickiness of neat honey, however, hinders its direct application for wound healing [56]. This limitation can be overcome by incorporating honey into a suitable carrier [56]. It is paramount that the formulation has a high honey load incorporated into an inert carrier that does not adversely affect the honey’s chemical composition and bioactivity. Moreover, the manufacturing process must also not affect the bioactivity profile of the honey [21]. The present study demonstrates that three different honey-loaded alginate-based formulations, namely a pre-gel solution as well as a wet sheet and also a dry sheet, all with a high honey loading, can be successfully prepared using sodium alginate as a carrier and calcium chloride as a crosslinking agent without negative impacts on the presence and concentration of monitored honey constituents.

While Manuka honey-loaded pre-gel solution formulations and also the equivalent wet sheets prepared in this study have been formulated before, to our knowledge this study is the first to report on alginate-based dry sheet honey formulations, which are characterised not only by a high honey loading, but also a large swelling index and relatively high tensile strength. The dry sheets can, therefore, be anticipated to be useful for the treatment of strongly weeping wounds and to be easier to handle. Due to their lower water content, they may also be less prone to microbial degradation compared to corresponding wet sheets and pre-gel solutions.

The present methodology yields pre-gel solution formulations with consistent characteristics irrespective of the type of honey loaded. Although the neat honeys themselves showed significant differences in moisture content and spreading behaviour, once formulated with sodium alginate into pre-gel solutions, there was no difference in moisture content as well as spreadability among the pre-gel solutions (Table 3 and Table 4). The viscosity of the pre-gel solution formulations was distinct at the two investigated temperatures (25 and 37 °C). However, similar viscosity was noted at a particular temperature (25 and 37 °C), which mimics the applications at room and body temperatures. This indicates that these formulations would behave consistently during their application at the respective clinically encountered temperatures. The pre-gel solution showed a higher degree of spreadability compared to corresponding neat honey (Table 5), which is advantageous to clinical application. Moreover, consistent thickness and length were obtained for both the dry sheets and wet sheets, which demonstrates the capability of the manufacturing process to produce honey-loaded gel sheets of defined and reproducible dimensions. Given their different swelling behaviours, distinct clinical applications can be envisaged for these sheets. With its much higher swelling index, the dry sheet can be anticipated to draw out more wound exudate and may, thus, be particularly suited to weeping wounds. Conversely, the wet sheet may exert a cooling effect on the skin due to its higher moisture content, which may also be advantageous in certain clinical circumstances, such as burns and inflamed wounds. With the MGO content in the Manuka honey-loaded pre-gel solutions, wet sheets, and dry sheets observed to be comparable to the neat Manuka honeys, it could be concluded that the MGO in the honeys was not negatively impacted by the range of manufacturing techniques employed in this study. Although the MGO content in the two WA Manuka honeys was found to be lower than that in the New Zealand Manuka honey used in this study (Table 9), in a subsequent study, the overall antibacterial activity of the three Manuka honeys was found to be almost identical. This finding correlates with the literature which emphasises that there are several factors, including low water activity, high osmotic pressure, low pH, low protein content and presence of glucose oxidase, some phenolic compounds, and bee-related enzymes, that all contribute to the overall antibacterial activity of non-peroxide honeys (i.e., Manuka honey) [11,13,26]. Similarly, in the case of peroxide honeys, the concentration of enzymatically formed H_2_O_2_ along with the aforementioned factors play an important role in their antibacterial activity [11,13,46]. The study also illustrated that the three formulation platforms are all suitable to incorporate different honeys to support clinical applications of honeys with different bioactivity profiles, thus offering more options compared to the current commercial honey-loaded products that mainly used the New Zealand Manuka honey.

In addition to the physicochemical properties, the monitoring of selected honey constituents by HPTLC analysis revealed that the respective compounds remained in the formulations at more than 97% of the levels found in the respective neat honeys (Table 10), which demonstrates that the manufacturing process did not lead to a significant decrease in these components. Furthermore, both dry and wet sheets contained almost the same amounts of components, which means that, with respect to these potentially bioactive constituents, the wet and dry sheets can be expected to exert similar clinical effects. It should be noted that the components of interest, including the water-soluble MGO and the HPTLC-monitored water-insoluble non-sugar constituents, were not lost throughout the stepwise formulation manufacture processes, indicating that these formulation platforms are suitable to prepare a range of honey-loaded products without compromising the honey’s bioactive constituents.

Moreover, the representative WA Jarrah and Manuka 2 honey-loaded formulations remained stable in terms of pH, moisture, dimensions (thickness and length), tensile strength, and swelling index when stored for 6 months at 5, 30, and 40 °C. The spreadability of neat honeys and pre-gel solution formulations stored at 30 and 40 °C increased by about 2% compared to the baseline and the samples that were kept at 5 °C. Monitored selected honey constituents remained unchanged which indicates that any potential bioactivities associated with these components will retain their therapeutic effect, for example when the formulations are applied to wounds and burns.

It is interesting, though not unexpected, to note that HMF was generated in samples stored at 30 and 40 °C, a finding in line with previous studies that have demonstrated an increase in HMF content in honey upon storage at medium (about 30 °C) or higher temperatures (above 50 °C) [52,53,54,57], in addition to storage in metallic containers and the honey’s floral sources itself which, next to temperature, were also identified as critical factors affecting HMF levels [54,57]. This finding means that storage conditions for these honey-loaded alginate-based formulations need to be carefully considered to minimize the increase in HMF levels.

## 5. Conclusions

In this study, five honeys (four WA honeys and one New Zealand comparator honey) were successfully formulated into three different alginate-based formulations (pre-gel solution, wet sheet, and dry sheet) by employing a simple and convenient stepwise processing method. Independent of the type of honey used in the manufacturing process, the resulting formulations feature high honey loadings and consistent physicochemical characteristics with the concentration of selected honey constituents also maintained at the same levels as found in the respective neat honeys. Storage stability data indicate that, except for MGO levels (where a slight decrease over time was noted) and spreadability of the pre-gel solution formulation (for which a slight increase over time was recorded), other physicochemical characteristics of the honey-loaded products and some of their non-sugar constituents retained their baseline levels. The fabrication method is not only capable of formulating products featuring a range of honeys besides Manuka honey, which is already available as commercial honey-loaded products, but also introduces a novel formulation (i.e., dry sheet) which is envisaged to be beneficial in certain clinical situations. It can, therefore, be concluded that the developed methodology is suitable to manufacture different honey-based formulations in a consistent and reproducible manner.

## Figures and Tables

**Table 1:** Honey samples including botanical origin.

Botanical Origin	Supplier, Year
WA Manuka Honey 1 (*Leptospermum scoparium*)	Hive and Wellness, 2019
WA Manuka Honey 2 (*Leptospermum scoparium*)	Manuka Life, 2019
WA Coastal Peppermint (*Agonis flexuosa*)	Margaret River Honey Company, 2019
WA Jarrah Honey (*Eucalyptus marginata*)	Hive and Wellness, 2019
New Zealand Manuka Honey (*Leptospermum scoparium)*	Hive and Wellness, 2018

**Table 2:** pH and colour profile of neat honeys and pre-gel solution formulations (*n* = 3, data represents mean ± SD).

Honey	Sample	pH	Colour at Pre- and Post-Filtration *	
			Pre	Post
Jarrah	Neat Honey	4.65 ± 0.01	541.00 ± 0.03	415.00 ± 0.03
	Pre-gel solution	5.34 ± 0.02	451.00 ± 0.03	221.00 ± 0.03
Coastal Peppermint	Neat Honey	4.75 ± 0.00	508.00 ± 0.03	320.00 ± 0.02
	Pre-gel solution	5.49 ± 0.01	390.00 ± 0.01	218.00 ± 0.01
WA Manuka 1	Neat Honey	4.56 ± 0.00	855.00 ± 0.03	445.00 ± 0.01
	Pre-gel solution	5.35 ± 0.00	525.00 ± 0.02	263.00 ± 0.03
WA Manuka 2	Neat Honey	4.65 ± 0.01	780.00 ± 0.01	545.00 ± 0.02
	Pre-gel solution	5.42 ± 0.00	523 ± 0.01	343 ± 0.00
NZ Manuka	Neat Honey	4.60 ± 0.00	1146 ± 0.03	715 ± 0.01
	Pre-gel solution	5.38 ± 0.00	696 ± 0.02	361 ± 0.03

* Filtration with a 0.7 µm disposable syringe filter.

**Table 3:** Moisture content of neat honeys and pre-gel solution formulations (*n* = 3, data represents mean ± SD).

Honey Type	Moisture Content (%)	
	Neat Honey	Pre-Gel Solution
Jarrah Honey	17.30 ± 0.10	49.00 ± 0.53
Coastal Peppermint Honey	18.00 ± 0.35	48.37 ± 0.15
WA Manuka Honey 1	18.75 ± 0.26	48.57 ± 0.06
WA Manuka Honey 2	19.58 ± 0.32	49.00 ± 0.00
NZ Manuka Honey	20.30 ± 0.10	48.63 ± 0.26

**Table 4:** Spreadability of neat honeys and pre-gel solution formulations (*n* = 3, data represents mean ± SD).

Honey Type	Spreadability (g·cm/s)	
	Neat Honey	Pre-Gel Solution
Jarrah Honey	334.81 ± 0.10	425.11 ± 0.10
Coastal Peppermint Honey	267.51 ± 0.33	425.09 ± 0.11
WA Manuka Honey 1	368.43 ± 0.46	425.11 ± 0.11
WA Manuka Honey 2	324.34 ± 0.22	424.84 ± 0.11
NZ Manuka Honey	336.96 ± 0.09	424.89 ± 0.11

**Table 5:** Viscosity of neat honeys and their respective pre-gel solution formulations (*n* = 3, data represents mean ± SD).

Honey Type	Neat Honey (mPa·s)		Pre-Gel Solutions (mPa·s)	
	Temperature (25 °C)	Temperature (37 °C)	Temperature (25 °C)	Temperature (37 °C)
WA Jarrah Honey	15,286.73 ± 0.75	3509.71 ± 0.79	2882.07 ± 0.68	2214.39 ± 0.75
WA Coastal Peppermint Honey	14,516.03 ± 0.67	3217.66 ± 0.81	2881.07 ± 0.68	2214.69 ± 0.73
WA Manuka Honey 1	3826.42 ± 0.66	2328.04 ± 0.78	2880.76 ± 0.59	2215.62 ± 0.69
WA Manuka Honey 2	6464.73 ± 0.71	2016.34 ± 0.77	2881.34 ± 0.59	2214.88 ± 0.71
NZ Manuka Honey	5355.28 ± 0.67	2561.36 ± 0.81	2882.22 ± 0.62	2214.79 ± 0.72

**Table 6:** Dimensions of honey-loaded wet and dry sheets (*n* = 3, data represents mean ± SD).

Sample	Thickness (mm)		Length (mm)	
	Wet Sheet	Dry Sheet	Wet Sheet	Dry Sheet
WA Jarrah Honey	2.01 ± 0.02	1.41 ± 0.02	94.24 ± 0.03	93.33 ± 0.02
WA Coastal Peppermint Honey	2.00 ± 0.01	1.39 ± 0.02	94.22 ± 0.02	93.32 ± 0.02
WA Manuka Honey 1	2.02 ± 0.01	1.40 ± 0.02	94.23 ± 0.02	93.29 ± 0.02
WA Manuka Honey 2	2.01 ± 0.02	1.39 ± 0.02	94.21 ± 0.02	93.29 ± 0.02
NZ Manuka Honey	2.02 ± 0.01	1.41 ± 0.02	94.19 ± 0.03	93.31 ± 0.02

**Table 7:** Tensile strength of honey-loaded wet and dry sheets (*n* = 3, data represents mean ± SD).

Honey Type	Tensile Strength (Pa)	
	Wet Sheet	Dry Sheet
Jarrah Honey	106.15 ± 0.28	192.25 ± 0.46
Coastal Peppermint Honey	100.11 ± 0.25	185.28 ± 0.62
WA Manuka Honey 1	108.65 ± 0.42	190.34 ± 0.56
WA Manuka Honey 2	109.31 ± 0.54	194.42 ± 0.62
NZ Manuka Honey	111.55 ± 0.38	200.45 ± 0.64

**Table 8:** Swelling Index of Dry Sheets (*n* = 3, data represents mean ± SD).

Sample (Dry Sheet)	Swelling Index (%) Measured at Different Time Points			
	10 min	20 min	30 min	After 45 min
WA Jarrah Honey	52.08 ± 0.12	57.8 ± 0.17	57.8 ± 0.17	57.8 ± 0.08
WA Coastal Peppermint Honey	52.04 ± 0.21	58.07 ± 0.22	58.06 ± 0.18	58.05 ± 0.09
WA Manuka Honey 1	52.07 ± 0.18	57.79 ± 0.20	57.72 ± 0.24	57.71 ± 0.07
WA Manuka Honey 2	52.06 ± 0.16	58.03 ± 0.19	58.04 ± 0.16	58.04 ± 0.07
NZ Manuka Honey	51.97 ± 0.18	57.86 ± 0.14	57.83 ± 0.10	57.82 ± 0.08

**Table 9:** MGO content in the neat Manuka honeys and corresponding honey-loaded formulations (*n* = 3, data represent mean ± SD).

Honey Type	MGO (mg/kg)			
	Neat Honey	Pre-Gel Solution	Wet Sheet	Dry Sheet
WA Manuka Honey 1	95.65 ± 1.4	94.89 ± 1.3	95.27 ± 1.4	95.19 ± 1.2
WA Manuka Honey 2	180.55 ± 1.2	180.65 ± 1.3	179.97 ± 1.4	180.32 ± 1.3
NZ Manuka Honey	350.47 ± 1.1	349.95 ± 1.4	350.07 ± 1.3	350.21 ± 1.3

**Table 10:** Peak area of selected bands of neat honeys and formulations (*n* = 3, data represents mean ± SD).

Honey	Weight (g)	Rf of Monitored Compound	Peak Area (AU × 10^−3^) per Band				Peak Area (AU × 10^−3^) per Sheet	
			Neat Honey	Pre-Gel Solution	Wet Sheet	Dry Sheet	Wet Sheet	Dry Sheet
WA Jarrah	1.01	0.20	5.2 ± 0.01	5.2 ± 0.03	5.1 ± 0.01	14.0 ± 0.04	128 ± 0.02	126 ± 0.01
		0.53	15.2 ± 0.02	15.2 ± 0.03	14.8 ± 0.05	40.8 ± 0.03	369 ± 0.01	368 ± 0.03
WA Coastal Peppermint	1.02	0.20	9.4 ± 0.04	9.4 ± 0.04	9.4 ± 0.04	26.1 ± 0.03	235 ± 0.03	235 ± 0.02
		0.53	22.9 ± 0.01	22.8 ± 0.02	22.7 ± 0.05	63.2 ± 0.04	570 ± 0.02	569 ± 0.04
WA Manuka 1	1.01	0.38	26.8 ± 0.02	26.7 ± 0.02	26.7 ± 0.06	74.0 ± 0.02	668 ± 0.03	667 ± 0.03
		0.53	6.8 ± 0.06	6.7 ± 0.06	6.7 ± 0.07	18.5 ± 0.03	168 ± 0.02	167 ± 0.02
WA Manuka 2	1.01	0.20	17.5 ± 0.05	17.2 ± 0.07	17.2 ± 0.04	47.6 ± 0.04	430 ± 0.03	429 ± 0.02
		0.38	23.6 ± 0.04	23.4 ± 0.01	23.4 ± 0.04	65.0 ± 0.02	586 ± 0.02	585 ± 0.03
NZ Manuka	1.02	0.32	13.7 ± 0.06	13.7 ± 0.04	13.3 ± 0.06	36.8 ± 0.01	332 ± 0.04	331 ± 0.02
		0.39	25.3 ± 0.02	25.0 ± 0.05	24.7 ± 0.07	67.8 ± 0.02	617 ± 0.02	617 ± 0.04

**Table 11:** pH, moisture content, and spreadability of Jarrah and WA Manuka honey 2 and their respective pre-gel solution formulations (*n* = 3, data represents mean ± SD).

Honey	Storage Temperature (°C)	Sample	pH		Moisture Content (%)		Spreadability (g·cm/s)	
			Baseline	6 Months	Baseline	6 Months	Baseline	6 Months
Jarrah	5	Neat Honey	4.62 ± 0.02	4.62 ± 0.03	17.88 ± 0.3	17.87 ± 0.2	334.77 ± 0.2	334.75 ± 0.2
		Pre-gel	5.30 ± 0.03	5.31 ± 0.03	48.98 ± 0.2	48.95 ± 0.3	425.15 ± 0.1	425.14 ± 0.2
	30	Neat Honey	4.61 ± 0.03	4.62 ± 0.02	17.87 ± 0.3	17.87 ± 0.2	336.77 ± 0.2	337.75 ± 0.2
		Pre-gel	5.29 ± 0.02	5.30 ± 0.04	48.89 ± 0.2	48.91 ± 0.3	427.15 ± 0.1	428.14 ± 0.3
	40	Neat Honey	4.61 ± 0.02	4.63 ± 0.03	17.89 ± 0.3	17.88 ± 0.2	338.77 ± 0.2	337.75 ± 0.2
		Pre-gel	5.29 ± 0.04	5.31 ± 0.03	48.86 ± 0.2	48.85 ± 0.3	426.15 ± 0.1	427.14 ± 0.3
WA Manuka 2	5	Neat Honey	4.63 ± 0.01	4.62 ± 0.02	19.45 ± 0.2	19.44 ± 0.2	325.47 ± 0.2	325.45 ± 0.2
		Pre-gel	5.40 ± 0.02	5.38 ± 0.02	49.08 ± 0.2	49.09 ± 0.3	424.93 ± 0.2	424.94 ± 0.1
	30	Neat Honey	4.61 ± 0.02	4.61 ± 0.03	19.44 ± 0.2	19.41 ± 0.2	324.47 ± 0.2	324.45 ± 0.2
		Pre-gel	5.38 ± 0.02	5.39 ± 0.03	49.08 ± 0.2	49.08 ± 0.3	424.93 ± 0.2	426.94 ± 0.1
	40	Neat Honey	4.60 ± 0.02	4.62 ± 0.03	19.43 ± 0.2	19.43 ± 0.2	325.47 ± 0.2	324.45 ± 0.3
		Pre-gel	5.38 ± 0.02	5.29 ± 0.02	49.08 ± 0.2	49.10 ± 0.3	424.93 ± 0.2	423.94 ± 0.2

**Table 12:** Dimension and tensile strength of Jarrah and WA Manuka honey 2 and their respective pre-gel solution formulations (*n* = 3, data represents mean ± SD).

Honey	Storage Temperature (°C)	Sample	Thickness (mm)		Length (mm)		Tensile Strength (Pa)	
			Baseline	6 Months	Baseline	6 Months	Baseline	6 Months
Jarrah	5	Wet	2.01 ± 0.02	2.02 ± 0.02	94.29 ± 0.02	94.27 ± 0.02	107.18 ± 0.31	106.89 ± 0.31
		Dry	1.41 ± 0.02	1.41 ± 0.03	93.32 ± 0.02	93.34 ± 0.02	193.33 ± 0.32	193.36 ± 0.32
	30	Wet	2.02 ± 0.02	2.00 ± 0.02	94.28 ± 0.02	94.27 ± 0.03	107.38 ± 0.21	107.89 ± 0.21
		Dry	1.41 ± 0.02	1.39 ± 0.03	93.31 ± 0.01	93.34 ± 0.02	193.83 ± 0.22	193.86 ± 0.22
	40	Wet	2.02 ± 0.02	2.00 ± 0.02	94.28 ± 0.02	94.26 ± 0.02	107.78 ± 0.21	108.07 ± 0.21
		Dry	1.40 ± 0.02	1.39 ± 0.03	93.30 ± 0.02	93.32 ± 0.02	194.33 ± 0.22	194.66 ± 0.26
WA Manuka 2	5	Wet	2.01 ± 0.02	2.01 ± 0.02	94.25 ± 0.02	94.21 ± 0.02	108.33 ± 0.32	108.31 ± 0.34
		Dry	1.39 ± 0.02	1.39 ± 0.02	93.28 ±0.02	93.27 ±0.03	194.65 ± 0.32	194.64 ± 0.32
	30	Wet	2.02 ± 0.01	2.01 ± 0.02	94.26 ± 0.02	94.23 ± 0.02	108.64 ± 0.22	108.66 ± 0.24
		Dry	1.39 ± 0.01	1.39 ± 0.02	93.27 ± 0.02	93.24 ± 0.03	194.85 ± 0.22	194.97 ± 0.22
	40	Wet	2.01 ± 0.02	2.01 ± 0.02	94.26 ± 0.02	94.22 ± 0.02	108.93 ± 0.22	108.41 ± 0.24
		Dry	1.40 ± 0.02	1.39 ± 0.02	93.26 ± 0.02	93.27 ± 0.01	194.95 ± 0.22	194.88 ± 0.22

**Table 13:** Swelling index of dry sheets of prepared with Jarrah and WA Manuka honey 2 (*n* = 3, data represents mean ± SD).

Duration	Time (min)	5 °C		30 °C		40 °C	
		WA Jarrah Honey	WA Manuka Honey 2	WA Jarrah Honey	WA Manuka Honey 2	WA Jarrah Honey	WA Manuka Honey 2
1 month	10	52.12 ± 0.15	54.52 ± 0.16	52.22 ± 0.17	54.32 ± 0.18	52.21 ± 0.17	54.22 ± 0.18
	20	55.57 ± 0.17	58.60 ± 0.19	55.55 ± 0.15	58.57 ± 0.19	55.56 ± 0.15	58.56 ± 0.19
	30	55.61 ± 0.15	58.63 ± 0.16	55.60 ± 0.15	58.60 ± 0.17	55.60 ± 0.15	58.59 ± 0.17
6 months	10	52.11 ± 0.17	54.51 ± 0.18	52.13 ± 0.17	54.31 ± 0.18	52.14 ± 0.17	54.21 ± 0.18
	20	55.56 ± 0.17	58.59 ± 0.19	55.57 ± 0.17	58.57 ± 0.19	55.57 ± 0.17	58.57 ± 0.19
	30	55.59 ± 0.17	58.61 ± 0.21	55.60 ± 0.17	58.61 ± 0.21	55.60 ± 0.17	58.60 ± 0.21

**Table 14:** Peak area of selected bands of Jarrah honey and WA Manuka honey 2 and their respective formulations (*n* = 3, data represents mean ± SD).

Honey	Storage Temperature (°C)	Sample	Weight (g)	Rf of Monitored Compound	Peak Area (AU × 10^−3^) per Band (±SD)		Peak Area (AU × 10^−3^) per Sheet (±SD)	
					Baseline	6 Months	Baseline	6 Months
WA Jarrah	5	Neat honey	1.01	0.20	5.3 ± 0.01	5.3 ± 0.01	N/A	N/A
				0.53	15.2 ± 0.02	15.2 ± 0.02	N/A	N/A
		Pre-gel	1.02	0.20	5.3 ± 0.03	5.3 ± 0.03	N/A	N/A
				0.53	15.2 ± 0.02	15.2 ± 0.02	N/A	N/A
		Wet sheet	1.01	0.20	5.2 ± 0.01	5.2 ± 0.01	130 ± 0.02	130 ± 0.02
				0.53	14.8 ± 0.03	14.8 ± 0.03	370 ± 0.02	370 ± 0.02
		Dry sheet	1.02	0.20	14.0 ± 0.04	14.0 ± 0.02	126 ± 0.01	126 ± 0.01
				0.53	40.8 ± 0.03	40.8 ± 0.02	367 ± 0.03	367 ± 0.03
	30	Neat honey	1.02	0.20	5.2 ± 0.02	5.2 ± 0.03	N/A	N/A
				0.53	15.2 ± 0.02	15.2 ± 0.02	N/A	N/A
		Pre-gel	1.01	0.20	5.2 ± 0.02	5.2 ± 0.02	N/A	N/A
				0.53	15.2 ± 0.02	15.2 ± 0.02	N/A	N/A
		Wet sheet	1.02	0.20	5.1 ± 0.01	5.1 ± 0.02	128 ± 0.02	128 ± 0.02
				0.53	14.8 ± 0.03	14.8 ± 0.03	370 ± 0.03	370 ± 0.03
		Dry sheet	1.02	0.20	14.1 ± 0.03	14.1 ± 0.03	127 ± 0.03	127 ± 0.03
				0.53	40.8 ± 0.03	40.8 ± 0.02	367 ± 0.02	367 ± 0.02
	40	Neat honey	1.01	0.20	5.2 ± 0.01	5.2 ± 0.01	N/A	N/A
				0.53	15.2 ± 0.01	15.2 ± 0.01	N/A	N/A
		Pre-gel	1.02	0.20	5.2 ± 0.03	5.2 ± 0.02	N/A	N/A
				0.53	15.2 ± 0.02	15.2 ± 0.02	N/A	N/A
		Wet sheet	1.01	0.20	5.1 ± 0.02	5.1 ± 0.02	128 ± 0.02	128 ± 0.02
				0.53	14.8 ± 0.02	14.8 ± 0.03	369 ± 0.02	369 ± 0.02
		Dry sheet	1.01	0.20	14.0 ± 0.03	14.0 ± 0.02	126 ± 0.03	126 ± 0.03
				0.53	40.8 ± 0.02	40.8 ± 0.02	367 ± 0.02	367 ± 0.02
WA Manuka 2	5	Neat honey	1.01	0.20	17.4 ± 0.03	17.4 ± 0.03	N/A	N/A
				0.38	23.5 ± 0.03	23.5 ± 0.02	N/A	N/A
		Pre-gel	1.02	0.20	17.3 ± 0.03	17.3 ± 0.03	N/A	N/A
				0.38	23.4 ± 0.02	23.4 ± 0.03	N/A	N/A
		Wet sheet	1.02	0.20	17.1 ± 0.03	17.1 ± 0.04	427 ± 0.02	427 ± 0.02
				0.38	23.3 ± 0.02	23.3 ± 0.03	582 ± 0.03	582 ± 0.03
		Dry sheet	1.01	0.20	47.5 ± 0.03	47.5 ± 0.03	428 ± 0.03	428 ± 0.03
				0.38	64.9 ± 0.02	64.9 ± 0.02	584 ± 0.03	584 ± 0.03
	30	Neat honey	1.02	0.20	17.4 ± 0.03	17.4 ± 0.03	N/A	N/A
				0.38	23.5 ± 0.03	23.5 ± 0.02	N/A	N/A
		Pre-gel	1.01	0.20	17.3 ± 0.03	17.3 ± 0.03	N/A	N/A
				0.38	23.4 ± 0.02	23.4 ± 0.02	N/A	N/A
		Wet sheet	1.02	0.20	17.2 ± 0.03	17.2 ± 0.04	428 ± 0.02	428 ± 0.02
				0.38	23.3 ± 0.02	23.3 ± 0.03	581 ± 0.03	581 ± 0.03
		Dry sheet	1.02	0.20	47.6 ± 0.03	47.6 ± 0.03	427 ± 0.03	427 ± 0.03
				0.38	65.0 ± 0.02	65.0 ± 0.03	585 ± 0.02	585 ± 0.02
	40	Neat honey	1.01	0.20	17.4 ± 0.03	17.4 ± 0.03	N/A	N/A
				0.38	23.5 ± 0.02	23.5 ± 0.02	N/A	N/A
		Pre-gel	1.02	0.20	17.3 ± 0.03	17.3 ± 0.03	N/A	N/A
				0.38	23.4 ± 0.03	23.4 ± 0.03	N/A	N/A
		Wet sheet	1.02	0.20	17.1 ± 0.03	17.1 ± 0.02	427 ± 0.03	427 ± 0.03
				0.38	23.3 ± 0.02	23.3 ± 0.03	582 ± 0.02	582 ± 0.02
		Dry sheet	1.02	0.20	47.5 ± 0.03	47.5 ± 0.03	427 ± 0.02	427 ± 0.02
				0.38	64.9 ± 0.03	64.9 ± 0.02	584 ± 0.02	584 ± 0.02

**Table 15:** MGO content data of WA Manuka honey 2 (*n* = 3, data represents mean ± SD).

Sample Type	Storage Temperature (°C)	MGO (mg/kg)					
		1 Month	2 Months	3 Months	4 Months	5 Months	6 Months
Neat honey	5	181.34 ± 1.2	181.65 ± 1.3	182.47 ± 1.2	181.82 ± 1.3	182.55 ± 1.2	182.55 ± 1.3
	30	180.12 ± 1.1	179.25 ± 1.2	178.54 ± 1.3	176.82 ± 1.3	175.55 ± 1.2	174.55 ± 1.2
	40	176.12 ± 1.2	174.45 ± 1.2	173.78 ± 1.2	171.88 ± 1.3	169.75 ± 1.2	168.65 ± 1.3
Pre-gel solution	5	182.34 ± 1.3	181.65 ± 1.3	182.47 ± 1.2	181.62 ± 1.3	182.65 ± 1.2	181.35 ± 1.3
	30	180.34 ± 1.2	179.85 ± 1.3	178.44 ± 1.2	177.73 ± 1.3	175.15 ± 1.2	174.35 ± 1.2
	40	177.14 ± 1.2	176.35 ± 1.3	173.34 ± 1.2	172.37 ± 1.3	169.19 ± 1.2	167.32 ± 1.2
Wet sheet	5	181.51 ± 1.1	181.55 ± 1.3	181.24 ± 1.2	181.42 ± 1.2	181.55 ± 1.2	181.54 ± 1.3
	30	180.75 ± 1.2	180.12 ± 1.3	179.42 ± 1.3	178.28 ± 1.3	177.72 ± 1.1	175.55 ± 1.1
	40	175.29 ± 1.2	174.53 ± 1.2	172.41 ± 1.2	171.22 ± 1.1	169.45 ± 1.2	168.33 ± 1.2
Dry sheet	5	181.67 ± 1.1	181.35 ± 1.3	181.34 ± 1.1	181.27 ± 1.2	181.22 ± 1.2	181.34 ± 1.2
	30	180.14 ± 1.2	179.25 ± 1.3	178.11 ± 1.2	177.32 ± 1.3	175.81 ± 1.1	174.55 ± 1.3
	40	175.61 ± 1.2	174.44 ± 1.3	173.33 ± 1.1	171.28 ± 1.2	169.27 ± 1.3	167.23 ± 1.2

## Data Availability

Not applicable.

## Supplementary Materials

The following supporting information can be downloaded at: https://www.mdpi.com/article/10.3390/pharmaceutics15051483/s1. Table S1: pH of neat honeys and pre-gel solution formulations (*n* = 3). Table S2: Moisture content (%) of neat honeys and pre-gel solution formulations. Table S3: Spreadability (g.cm/sec) of neat honeys and pre-gel solution formulations. Table S4: Thickness (mm) of wet and dry sheet. Table S5: Length (mm) of wet and dry sheet. Table S6: Tensile strength of honey-loaded wet and dry sheets. Table S7: Swelling Index of Dry Sheets at 5 °C. Table S8: Swelling Index of Dry Sheets at 30 °C. Table S9: Swelling Index of Dry Sheets at 40 °C. Table S10: Peak area of selected bands of Jarrah honey and WA Manuka honey 2 and their respective formulations. Table S11: Peak area of selected bands in wet and dry sheets of Jarrah and WA Manuka honey. Figure S1: Jarrah (JAR) honey—red box indicates the monitored bands at Rf 0.20 and 0.53; Track 1—4,5,7-trihydroxyflavone (internal standard), Track 2—JAR honey extract (system suitability test), Tracks 3–8—JAR neat honey extract collected at 1–6 months storage at 5 °C, Tracks 9–14—JAR neat honey extract collected at 1–6 months storage at 30 °C, and Tracks 15–20—JAR neat honey extract collected at 1–6 months storage at 40 °C; image taken at 366 nm. Figure S2: JAR honey—red box indicates the monitored bands at Rf 0.20 and 0.53; Track 1—4,5,7-trihydroxyflavone (internal standard), Track 2—JAR honey extract (system suitability test), Tracks 3–8—JAR pre-gel solution extract collected at 1–6 months storage at 5 °C, Tracks 9–14—JAR pre-gel solution extract collected at 1–6 months storage at 30 °C, and Tracks 15–20—JAR pre-gel solution extract collected at 1–6 months storage at 40 °C. image taken at 366 nm. Figure S3: JAR honey—red box indicates the monitored bands at Rf 0.20 and 0.53; Track 1—4,5,7-trihydroxyflavone (internal standard), Track 2—JAR honey extract (system suitability test), Tracks 3–8—JAR wet sheet extract collected at 1–6 months storage at 5 °C, Tracks 9–14—JAR wet sheet extract collected at 1–6 months storage at 30 °C, and Tracks 15–20—JAR wet sheet extract collected at 1–6 months storage at 40 °C; image taken at 366 nm. Figure S4: JAR honey—red box indicates the monitored bands at Rf 0.20 and 0.53; Track 1—4,5,7-trihydroxyflavone (internal standard), Track 2—JAR honey extract (system suitability test), Tracks 3–8—JAR dry sheet extract collected at 1–6 months storage at 5 °C, Tracks 9–14—JAR dry sheet extract collected at 1–6 months storage at 30 °C, and Tracks 15–20—JAR dry sheet extract collected at 1–6 months storage at 40 °C; image taken at 366 nm. Figure S5: WA Manuka 2 (WAM2) honey—red box indicates the monitored bands at Rf 0.20 and 0.38; Track 1—4,5,7-trihydroxyflavone (internal standard), Track 2—WAM 2 honey extract (system suitability test), Tracks 3–8—WAM2 neat honey extract collected at 1–6 months storage at 5 °C, Tracks 9–14—WAM2 neat honey extract collected at 1–6 months storage at 30 °C, and Tracks 15–20—WAM2 neat honey extract collected at 1–6 months storage at 40 °C; image taken at 366 nm. Figure S6: WAM2 honey—red box indicates the monitored bands at Rf 0.20 and 0.38; Track 1—4,5,7-trihydroxyflavone (internal standard), Track 2—WAM 2 honey extract (system suitability test), Tracks 3–8—WAM2 pre-gel solution extract collected at 1–6 months storage at 5 °C, Tracks 9–14—WAM2 pre-gel solution extract collected at 1–6 months storage at 30 °C, and Tracks 15–20—WAM2 pre-gel solution extract collected at 1–6 months storage at 40 °C; image taken at 366 nm. Figure S7: WAM2 honey—red box indicates the monitored bands at Rf 0.20 and 0.38; Track 1—4,5,7-trihydroxyflavone (internal standard), Track 2—WAM 2 honey extract (system suitability test), Tracks 3–8—WAM2 wet sheet extract collected at 1–6 months storage at 5 °C, Tracks 9–14—WAM2 wet sheet extract collected at 1–6 months storage at 30 °C, and Tracks 15–20—WAM2 wet sheet extract collected at 1–6 months storage at 40 °C; image taken at 366 nm. Figure S8: WAM2 honey—red box indicates the monitored bands at Rf 0.20 and 0.38; Track 1—4,5,7-trihydroxyflavone (internal standard), Track 2- WAM 2 honey extract (system suitability test), Tracks 3–8—WAM2 dry sheet extract collected at 1–6 months storage at 5 °C, Tracks 9–14—WAM2 dry sheet extract collected at 1–6 months storage at 30 °C, and Tracks 15–20—WAM2 dry sheet extract collected at 1–6 months storage at 40 °C; image taken at 366 nm. Figure S9: Absorbance spectra of HMF aqueous solution (grey line) and honey extract (blue line).
