# Supplementary material for: Design, Preparation, and Physicochemical Characterisation of Alginate-Based Honey-Loaded Topical Formulations
Source: Pharmaceutics. 2023 May 12;15(5):1483. doi: 10.3390/pharmaceutics15051483 (PMC10223922; doi:10.3390/pharmaceutics15051483)
Supplement: Supplementary file 1 [file pharmaceutics-15-01483-s001.zip › pharmaceutics-2358139-supplementary.pdf]

Supplementary Material

Design, Preparation, and Physicochemical Characterisation of Alginate-Based Honey-Loaded Topical Formulations

Md Lokman Hossain [1], Lee Yong Lim [1], Katherine Hammer [2,3], Dhanushka Hettiarachchi [1] and Cornelia Locher [1,3,*]

[1] Division of Pharmacy, School of Allied Health, University of Western Australia, Crawley WA 6009, Australia; mdlokman.hossain@research.uwa.edu.au (M.L.H.); lee.lim@uwa.edu.au (L.Y.L.); dhanushka.hettiarachchi@outlook.com (D.H.)

[2] School of Biomedical Sciences, University of Western Australia, Crawley WA 6009, Australia; katherine.hammer@uwa.edu.au (K.H.)

[3] Cooperative Research Centre for Honey Bee Products Limited, 128 Yanchep Beach Road, Yanchep, WA 6035, Australia

* Correspondence: connie.locher@uwa.edu.au

Table S1. pH of neat honeys and pre-gel solution formulations (n=3, data represents mean ± SD).

Honey	Storage temperature (°C)	Sample	pH					
			1 month	2 months	3 months	4 months	5 months	6 months
Jarrah	5	Neat Honey	4.61±0.02	4.60±0.03	4.61±0.02	4.61±0.03	4.61±0.02	4.62±0.03
		Pre-gel solution	5.29±0.03	5.28±0.03	5.30±0.03	5.31±0.04	5.31±0.03	5.31±0.03
	30	Neat Honey	4.60±0.03	4.61±0.03	4.59±0.03	4.60±0.02	4.61±0.02	4.62±0.02
		Pre-gel solution	5.29±0.02	5.30±0.03	5.29±0.02	5.29±0.02	5.29±0.02	5.30±0.04
	40	Neat Honey	4.62±0.02	4.61±0.03	4.62±0.02	4.61±0.03	4.62±0.02	4.63±0.03
		Pre-gel solution	5.30±0.04	5.29±0.03	5.29±0.03	5.31±0.04	5.30±0.03	5.31±0.03
WA Manuka 2	5	Neat Honey	4.63±0.01	4.62±0.03	4.61±0.02	4.63±0.03	4.62±0.03	4.62±0.02
		Pre-gel solution	5.40±0.02	5.39±0.03	5.39±0.04	5.41±0.3	5.40±0.04	5.38±0.02
	30	Neat Honey	4.61±0.02	4.60±0.03	4.59±0.03	4.59±0.03	4.60±0.03	4.61±0.03
		Pre-gel solution	5.38±0.02	5.39±0.03	5.39±0.04	5.39±0.3	5.40±0.04	5.39±0.03
	40	Neat Honey	4.63±0.02	4.62±0.03	4.61±0.02	4.62±0.03	4.61±0.03	4.62±0.03
		Pre-gel solution	5.38±0.02	5.40±0.03	5.40±0.04	5.41±0.3	5.40±0.03	5.29±0.02

Table S2. Moisture content (%) of neat honeys and pre-gel solution formulations (n=3, data represents mean ± SD).

Honey	Storage temperature (°C)	Sample	Moisture content (%)					
			1 month	2 months	3 months	4 months	5 months	6 months
Jarrah	5	Neat Honey	17.85±0.3	17.87±0.2	17.88±0.1	17.87±0.2	17.87±0.3	17.87±0.2
		Pre-gel solution	48.98±0.2	48.96±0.1	48.97±0.2	48.96±0.1	48.95±0.2	48.95±0.3
	30	Neat Honey	17.87±0.3	17.86±0.2	17.87±0.1	17.86±0.2	17.86±0.3	17.87±0.2
		Pre-gel solution	48.86±0.2	48.87±0.1	48.87±0.2	48.88±0.1	48.89±0.2	48.91±0.3
	40	Neat Honey	17.89±0.3	17.87±0.2	17.86±0.1	17.87±0.2	17.88±0.3	17.88±0.2
		Pre-gel solution	48.85±0.2	48.84±0.1	48.84±0.2	48.85±0.1	48.84±0.2	48.85±0.3
WA Manuka 2	5	Neat Honey	19.45±0.2	19.41±0.2	19.44±0.1	19.43±0.2	19.42±0.3	19.44±0.2
		Pre-gel solution	49.08±0.2	49.08±0.1	49.07±0.2	49.10±0.3	49.09±0.2	49.09±0.3
	30	Neat Honey	19.44±0.2	19.41±0.2	19.42±0.1	19.41±0.2	19.42±0.3	19.41±0.2
		Pre-gel solution	49.08±0.2	49.09±0.1	49.08±0.2	49.10±0.3	49.07±0.2	49.08±0.3
	40	Neat Honey	19.43±0.2	19.42±0.2	19.44±0.1	19.43±0.2	19.42±0.3	19.43±0.2
		Pre-gel solution	49.08±0.2	49.09±0.1	49.10±0.2	49.11±0.3	49.11±0.2	49.10±0.3

Table S3. Spreadability (g.cm/sec) of neat honeys and pre-gel solution formulations (n=3, data represents mean ± SD).

Honey	Storage temperature (°C)	Sample	Spreadability (g.cm/sec)					
			1 month	2 months	3 months	4 months	5 months	6 months
Jarrah	5	Neat Honey	334.77±0.2	334.76±0.2	334.74±0.1	334.73±0.2	334.74±0.2	334.75±0.2
		Pre-gel solution	425.15±0.1	425.14±0.2	425.14±0.1	425.13±0.2	425.13±0.1	425.14±0.2
	30	Neat Honey	336.77±0.2	336.76±0.2	337.74±0.1	337.73±0.2	337.72±0.2	337.75±0.2
		Pre-gel solution	427.15±0.1	427.14±0.2	428.14±0.1	427.13±0.2	428.13±0.1	428.14±0.3
	40	Neat Honey	338.77±0.2	338.76±0.2	337.74±0.1	337.73±0.2	337.72±0.2	337.75±0.2
		Pre-gel solution	427.15±0.1	427.14±0.2	426.14±0.1	426.13±0.2	427.13±0.1	427.14±0.3
WA Manuka 2	5	Neat Honey	325.47±0.2	325.46±0.3	325.45±0.2	325.47±0.3	325.44±0.2	325.45±0.2
		Pre-gel solution	424.93± 0.2	424.94± 0.1	424.93± 0.2	424.94± 0.1	424.95± 0.3	424.94± 0.1
	30	Neat Honey	35.47±0.2	324.46±0.3	324.45±0.2	324.47±03	324.44±0.2	324.45±0.2
		Pre-gel solution	425.93± 0.2	426.94± 0.1	426.93± 0.2	426.94± 0.1	426.95± 0.1	426.94± 0.1
	40	Neat Honey	325.47±0.2	324.46±0.3	324.45±0.1	324.47±03	324.44±0.2	324.45±0.3
		Pre-gel solution	424.93± 0.2	423.94± 0.1	423.93± 0.2	423.94± 0.1	423.95± 0.3	423.94± 0.2

Table S4. Thickness (mm) of wet and dry sheet (n=3, data represents mean ± SD).

Honey	Storage temperature (°C)	Sample	Thickness (mm)					
			1 month	2 months	3 months	4 months	5 months	6 months
Jarrah	5	Wet	2.01±0.02	2.02±0.02	2.00±0.03	2.03±0.02	2.01±0.01	2.02±0.02
		Dry	1.41±0.02	1.41±0.02	1.40±0.02	1.39±0.01	1.40±0.02	1.41±0.03
	30	Wet	2.02±0.02	2.02±0.02	2.01±0.03	2.01±0.02	2.01±0.01	2.00±0.02
		Dry	1.41±0.02	1.40±0.01	1.40±0.02	1.39±0.01	1.39±0.02	1.39±0.03
	40	Wet	2.02±0.02	2.01±0.02	2.01±0.03	2.01±0.02	2.00±0.01	2.00±0.02
		Dry	1.40±0.02	1.40±0.01	1.39±0.02	1.39±0.01	1.39±0.02	1.39±0.03
WA Manuka 2	5	Wet	2.01±0.02	2.01±0.02	2.02±0.01	2.02±0.02	2.02±0.01	2.01±0.02
		Dry	1.39±0.02	1.40±0.02	1.39±0.03	1.40±0.02	1.40±0.03	1.39±0.02
	30	Wet	2.02±0.01	2.02±0.02	2.02±0.01	2.01±0.02	2.01±0.01	2.01±0.02
		Dry	1.39±0.01	1.40±0.01	1.39±0.02	1.40±0.02	1.40±0.03	1.39±0.02
	40	Wet	2.01±0.02	2.00±0.02	2.00±0.01	2.00±0.02	2.01±0.01	2.01±0.02
		Dry	1.40±0.02	1.40±0.01	1.39±0.01	1.40±0.02	1.40±0.03	1.39±0.02

Table S5. Length (mm) of wet and dry sheet (n=3, data represents mean ± SD).

Honey	Storage temperature (°C)	Sample	Length (mm)					
			1 month	2 months	3 months	4 months	5 months	6 months
Jarrah	5	Wet	94.29±0.02	94.27±0.03	94.26±0.02	94.25±0.03	94.26±0.03	94.27±0.02
		Dry	93.32 ±0.02	93.31 ±0.03	93.34 ±0.02	93.34 ±0.02	93.33 ±0.03	93.34 ±0.02
	30	Wet	94.28±0.02	94.26±0.02	94.27±0.03	94.25±0.03	94.27±0.03	94.27±0.03
		Dry	93.31 ±0.01	93.30 ±0.02	93.33 ±0.02	93.32 ±0.03	93.31 ±0.03	93.34 ±0.02
	40	Wet	94.28±0.02	94.27±0.03	94.28±0.02	94.26±0.02	94.26±0.03	94.26±0.02
		Dry	93.30 ±0.02	93.30 ±0.01	93.32 ±0.02	93.33 ±0.01	93.33 ±0.03	93.32 ±0.02
WA Manuka 2	5	Wet	94.25±0.02	94.23±0.02	94.23±0.01	94.24±0.03	94.24±0.02	94.21±0.02
		Dry	93.28 ±0.02	93.27 ±0.02	93.27 ±0.02	93.28 ±0.02	93.27 ±0.02	93.27 ±0.03
	30	Wet	94.26±0.02	94.24±0.02	94.24±0.01	94.24±0.03	94.25±0.02	94.23±0.02
		Dry	93.27 ±0.02	93.26 ±0.02	93.25 ±0.02	93.27 ±0.02	93.27 ±0.02	93.24±0.03
	40	Wet	94.26±0.02	94.24±0.03	94.23±0.01	94.254±0.02	94.25±0.02	94.22±0.02
		Dry	93.26 ±0.02	93.25 ±0.02	93.26 ±0.01	93.27 ±0.02	93.27 ±0.02	93.27 ±0.01

Table S6. Tensile strength of honey-loaded wet and dry sheets (n=3, data represents mean ± SD).

Honey	Storage temperature (°C)	Sample	Tensile strength (Pa)					
			1 month	2 months	3 months	4 months	5 months	6 months
Jarrah	5	Wet	107.18±0.31	106.75±0.25	106.85±0.26	106.65±0.28	106.76±0.29	106.69±0.31
		Dry	193.33±0.32	193.35±0.34	193.28±0.31	193.27±0.35	193.41±0.35	193.36±0.32
	30	Wet	107.38±0.21	107.35±0.25	107.37±0.26	107.45±0.28	107.76±0.29	107.89±0.21
		Dry	193.83±0.22	193.95±0.24	194.09±0.21	194.17±0.25	194.41±0.25	195.86±0.22
	40	Wet	107.78±0.21	107.95±0.25	108.15±0.26	108.25±0.28	108.86±0.29	108.87±0.21
		Dry	194.33±0.22	194.35±0.24	194.58±0.21	195.16±0.25	195.47±0.25	195.66±0.26
WA Manuka 2	5	Wet	108.33±0.32	108.31±0.34	108.31±0.32	108.31±0.29	108.31±0.34	108.31±0.34
		Dry	194.65±0.32	194.62±0.32	194.72±0.32	194.32±0.32	194.75±0.32	194.64±0.32
	30	Wet	108.64±0.22	108.86±0.24	108.93±0.22	109.11±0.29	109.43±0.24	109.66±0.24
		Dry	194.85±0.22	194.95±0.22	195.32±0.22	195.52±0.22	195.65±0.22	195.97±0.22
	40	Wet	108.93±0.22	108.99±0.24	109.09±0.22	109.11±0.29	109.22±0.24	109.41±0.24
		Dry	194.95±0.22	195.22±0.22	195.32±0.22	195.42±0.22	195.77±0.32	195.88±0.22

Table S7. Swelling Index of Dry Sheets at 5°C (n=3, data represents mean ± SD).

Duration of stability study (month)	Time (min)	Sample (Dry sheet)	
		WA Jarrah Honey	WA Manuka Honey 2
	10	52.12±0.15	54.52±0.16
1	20	55.57±0.17	58.60±0.19
	30	55.61±0.15	58.63±0.16
	10	52.11±0.12	54.51±0.16
2	20	55.58±0.17	58.59±0.16
	30	55.59±0.15	58.62±0.16
	10	52.12±0.12	54.51±0.19
3	20	55.61±0.17	58.59±0.19
	30	55.62±0.15	58.62±0.19
	10	52.09±0.12	54.52±0.16
4	20	55.55±0.18	58.53±0.16
	30	55.58±0.15	58.61±0.16
	10	52.08±0.12	54.51±0.19
5	20	55.57±0.17	58.52±0.19
	30	55.59±0.15	58.63±0.16
	10	52.11±0.17	54.51±0.18
6	20	55.56±0.17	58.59±0.19
	30	55.59±0.17	58.61±0.21

Table S8. Swelling Index of Dry Sheets at 30°C (n=3, data represents mean ± SD).

Duration of stability study (month)	Time (min)	Sample (Dry sheet)	
		WA Jarrah Honey	WA Manuka Honey 2
	10	52.22±0.17	54.32±0.18
1	20	55.55±0.15	58.57±0.19
	30	55.60±0.15	58.60±0.17
	10	52.21±0.12	54.31±0.16
2	20	55.56±0.17	58.56±0.16
	30	55.59±0.15	58.60±0.16
	10	52.12±0.12	54.31±0.19
3	20	55.56±0.17	58.57±0.17
	30	55.59±0.17	58.59±0.19
	10	52.11±0.15	54.32±0.16
4	20	55.56±0.18	58.55±0.16
	30	55.59±0.15	58.60±0.16
	10	52.10±0.15	54.31±0.17
5	20	55.55±0.12	58.55±0.19
	30	55.58±0.15	58.60±0.16
	10	52.13±0.17	54.31±0.18
6	20	55.57±0.17	58.57±0.19
	30	55.60±0.17	58.61±0.21

Table S9. Swelling Index of Dry Sheets at 40°C (n=3, data represents mean ± SD).

Duration of stability study (month)	Time (min)	Sample (Dry sheet)	
		WA Jarrah Honey	WA Manuka Honey 2
	10	52.21±0.17	54.22±0.18
1	20	55.56±0.15	58.56±0.19
	30	55.60±0.15	58.59±0.17
	10	52.22±0.12	54.21±0.16
2	20	55.56±0.17	58.55±0.16
	30	55.60±0.15	58.60±0.16
	10	52.17±0.12	54.21±0.19
3	20	55.57±0.17	58.56±0.17
	30	55.59±0.17	58.59±0.19
	10	52.21±0.15	54.22±0.16
4	20	55.56±0.18	58.54±0.16
	30	55.59±0.15	58.60±0.16
	10	52.14±0.15	54.21±0.17
5	20	55.56±0.12	58.55±0.19
	30	55.59±0.15	58.60±0.16
	10	52.14±0.17	54.21±0.18
6	20	55.57±0.17	58.57±0.19
	30	55.60±0.17	58.60±0.21

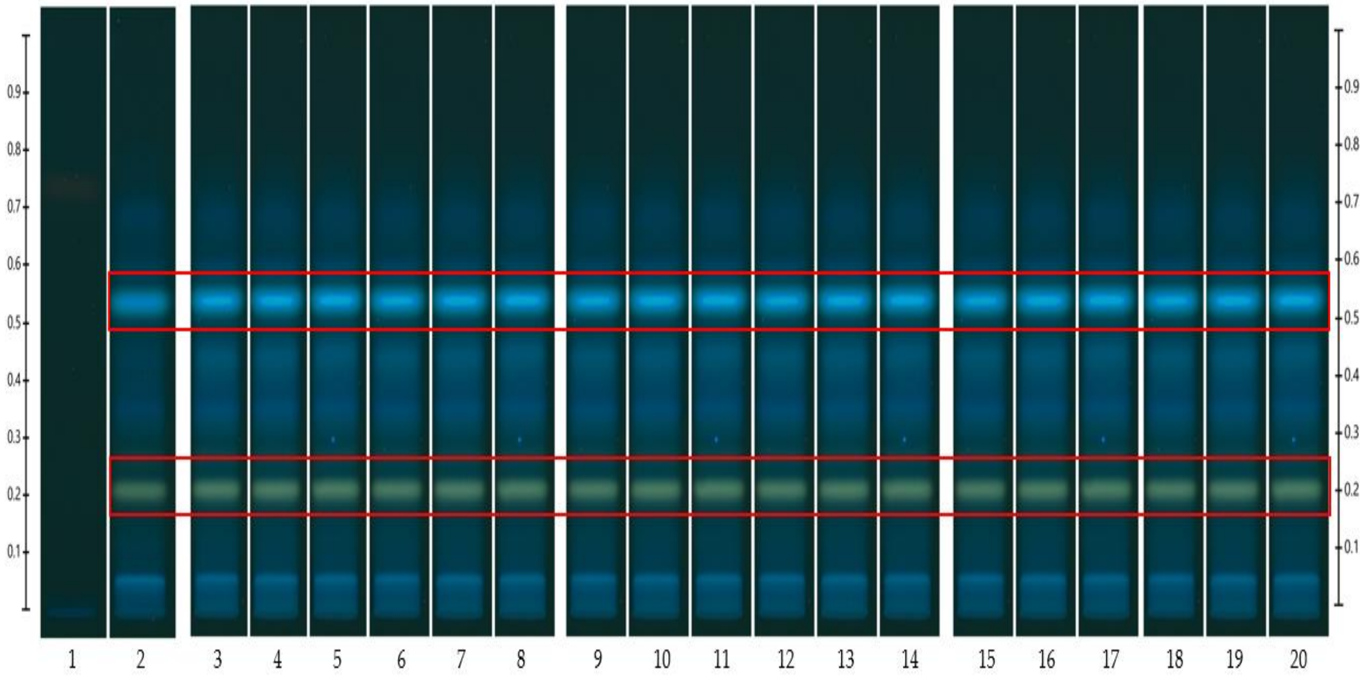

Figure S1. Jarrah (JAR) honey - Red box indicates the monitored bands at Rf 0.20 and 0.53; Track 1 - 4,5,7-trihydroxyflavone (internal standard), Track 2- JAR honey extract (system suitability test), Tracks 3-8 - JAR neat honey extract collected at 1-6 months storage at 5°C, Tracks 9-14 – JAR neat honey extract collected at 1-6 months storage at 30°C, Tracks 15-20 – JAR neat honey extract collected at 1-6 months storage at 40°C; Image taken at 366 nm.

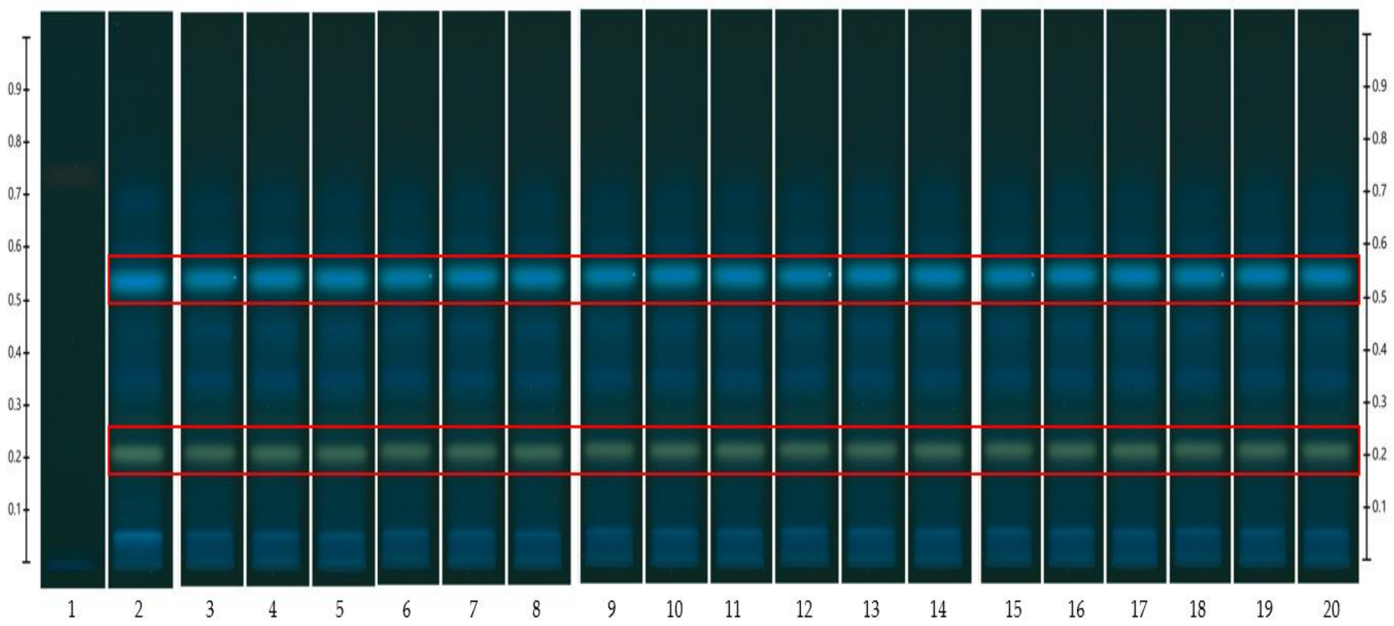

Figure S2. JAR honey - Red box indicates the monitored bands at Rf 0.20 and 0.53; Track 1 - 4,5,7-trihydroxyflavone (internal standard), Track 2- JAR honey extract (system suitability test), Tracks 3-8 - JAR pre-gel solution extract collected at 1-6 months storage at 5°C, Tracks 9-14 – JAR pre-gel solution extract collected at 1-6 months storage at 30°C, Tracks 15-20 – JAR pre-gel solution extract collected at 1-6 months storage at 40°C; Image taken at 366 nm.

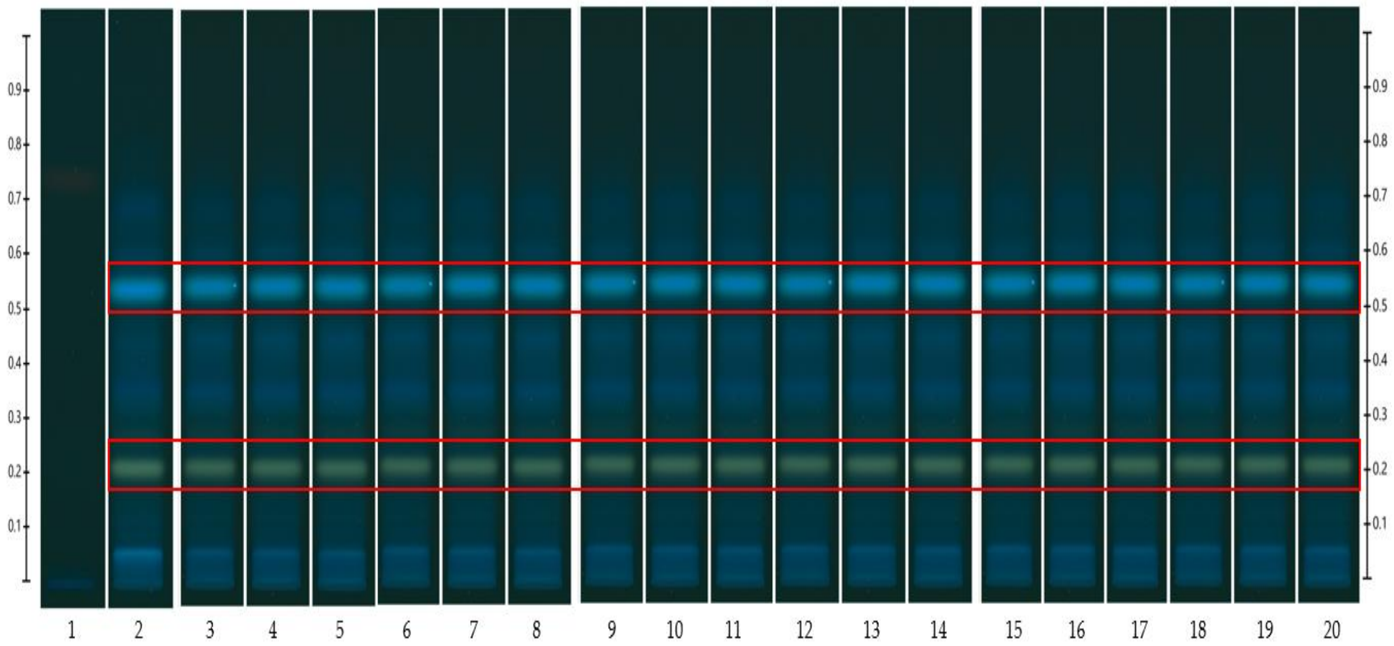

Figure S3. JAR honey - Red box indicates the monitored bands at Rf 0.20 and 0.53; Track 1 - 4,5,7-trihydroxyflavone (internal standard), Track 2- JAR honey extract (system suitability test), Tracks 3-8 - JAR wet sheet extract collected at 1-6 months storage at 5°C, Tracks 9-14 – JAR wet sheet extract collected at 1-6 months storage at 30°C, Tracks 15-20 – JAR wet sheet extract collected at 1-6 months storage at 40°C; Image taken at 366 nm.

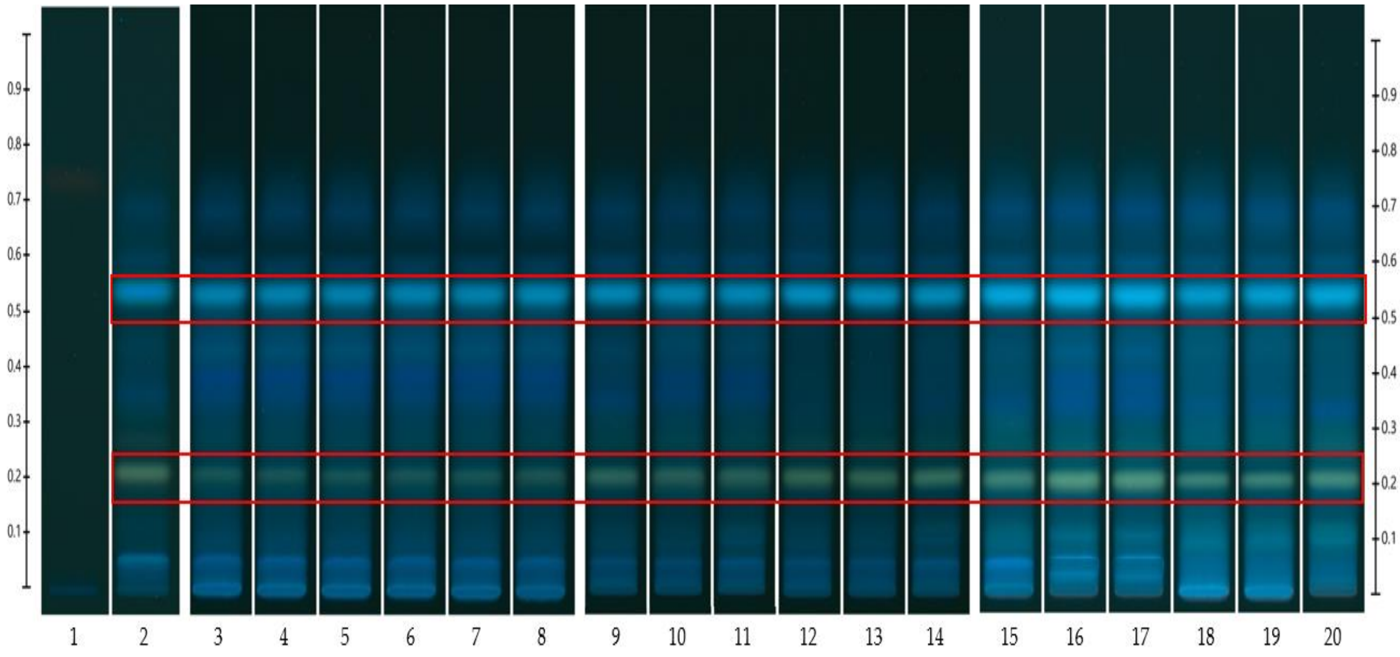

Figure S4. JAR honey - Red box indicates the monitored bands at Rf 0.20 and 0.53; Track 1 - 4,5,7-trihydroxyflavone (internal standard), Track 2- JAR honey extract (system suitability test), Tracks 3-8 - JAR dry sheet extract collected at 1-6 months storage at 5°C, Tracks 9-14 – JAR dry sheet extract collected at 1-6 months storage at 30°C, Tracks 15-20 – JAR dry sheet extract collected at 1-6 months storage at 40°C; Image taken at 366 nm.

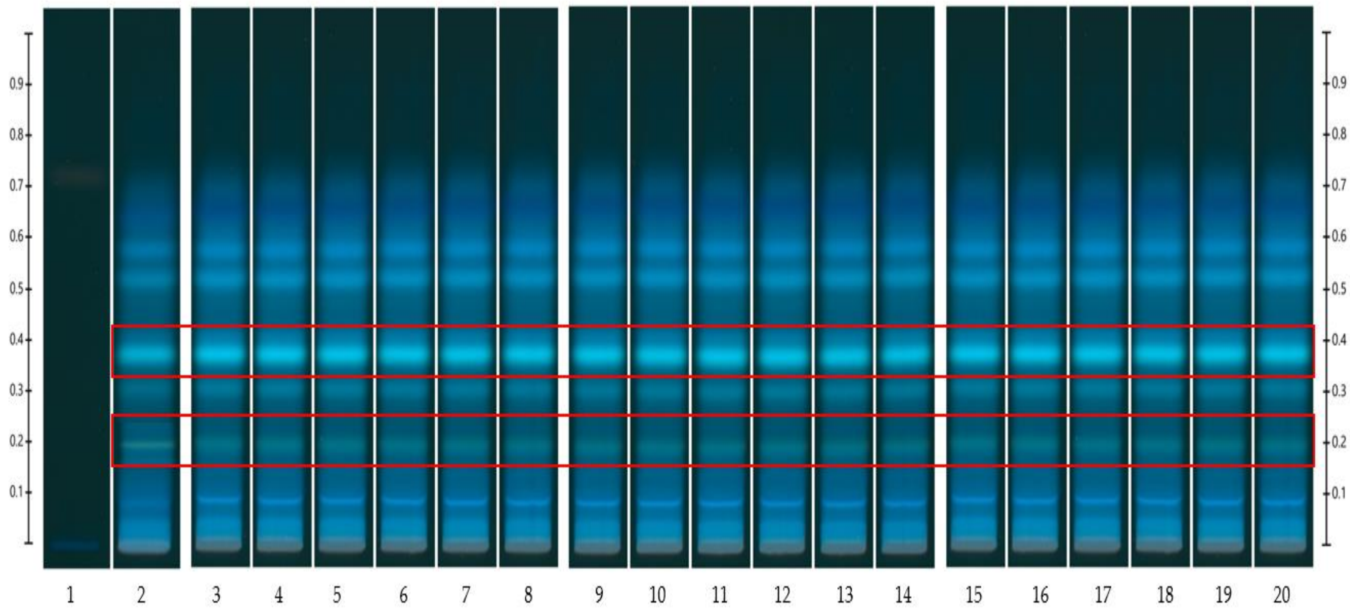

Figure S5. WA Manuka 2 (WAM2) honey - Red box indicates the monitored bands at Rf 0.20 and 0.38; Track 1 - 4,5,7-trihydroxyflavone (internal standard), Track 2- WAM 2 honey extract (system suitability test), Tracks 3-8 - WAM2 neat honey extract collected at 1-6 months storage at 5°C, Tracks 9-14 – WAM2 neat honey extract collected at 1-6 months storage at 30°C, Tracks 15-20 – WAM2 neat honey extract collected at 1-6 months storage at 40°C; Image taken at 366 nm.

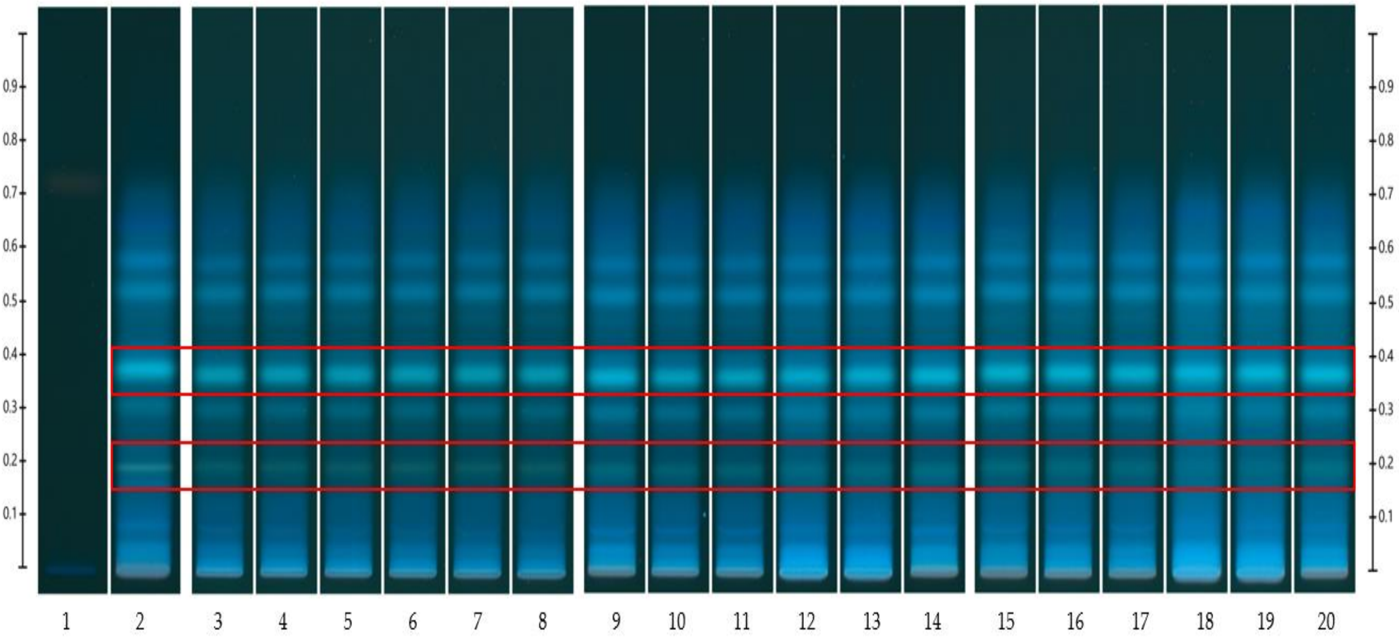

Figure S6. WAM2 honey - Red box indicates the monitored bands at Rf 0.20 and 0.38; Track 1 - 4,5,7-trihydroxyflavone (internal standard), Track 2- WAM 2 honey extract (system suitability test), Tracks 3-8 - WAM2 pre-gel solution extract collected at 1-6 months storage at 5°C, Tracks 9-14 – WAM2 pre-gel solution extract collected at 1-6 months storage at 30°C, Tracks 15-20 – WAM2 pre-gel solution extract collected at 1-6 months storage at 40°C; Image taken at 366 nm.

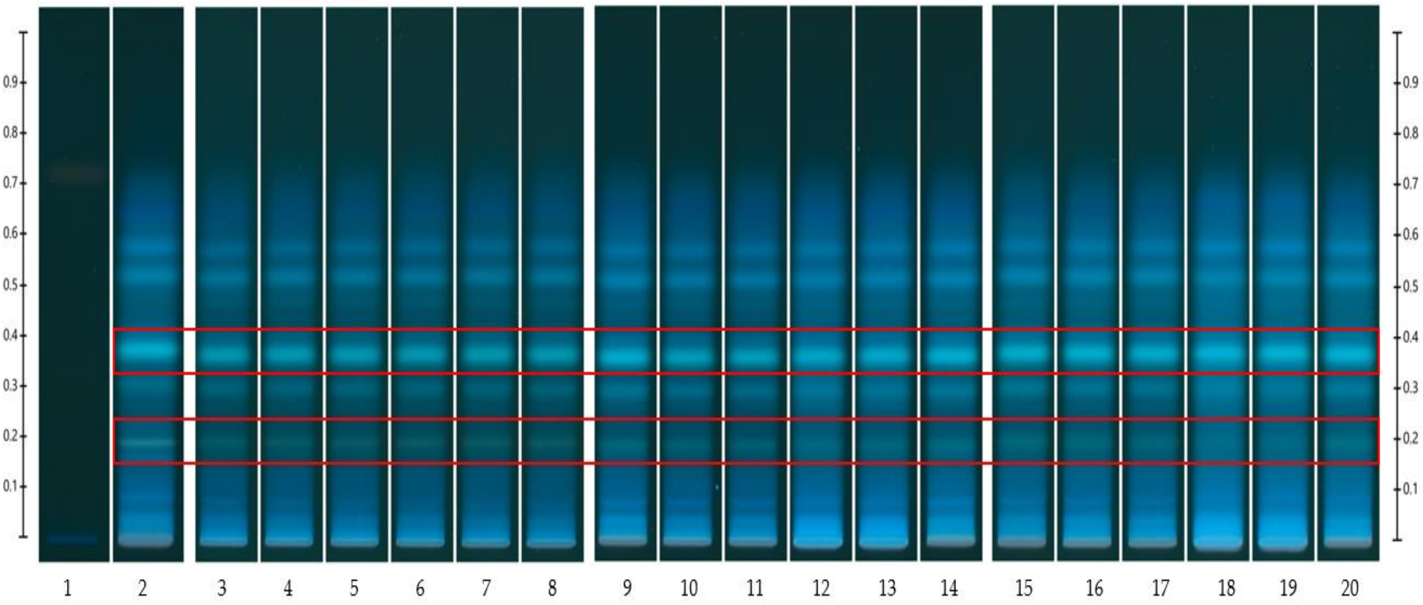

Figure S7. WAM2 honey - Red box indicates the monitored bands at Rf 0.20 and 0.38; Track 1 - 4,5,7-trihydroxyflavone (internal standard), Track 2- WAM 2 honey extract (system suitability test), Tracks 3-8 - WAM2 wet sheet extract collected at 1-6 months storage at 5°C, Tracks 9-14 – WAM2 wet sheet extract collected at 1-6 months storage at 30°C, Tracks 15-20 – WAM2 wet sheet extract collected at 1-6 months storage at 40°C; Image taken at 366 nm.

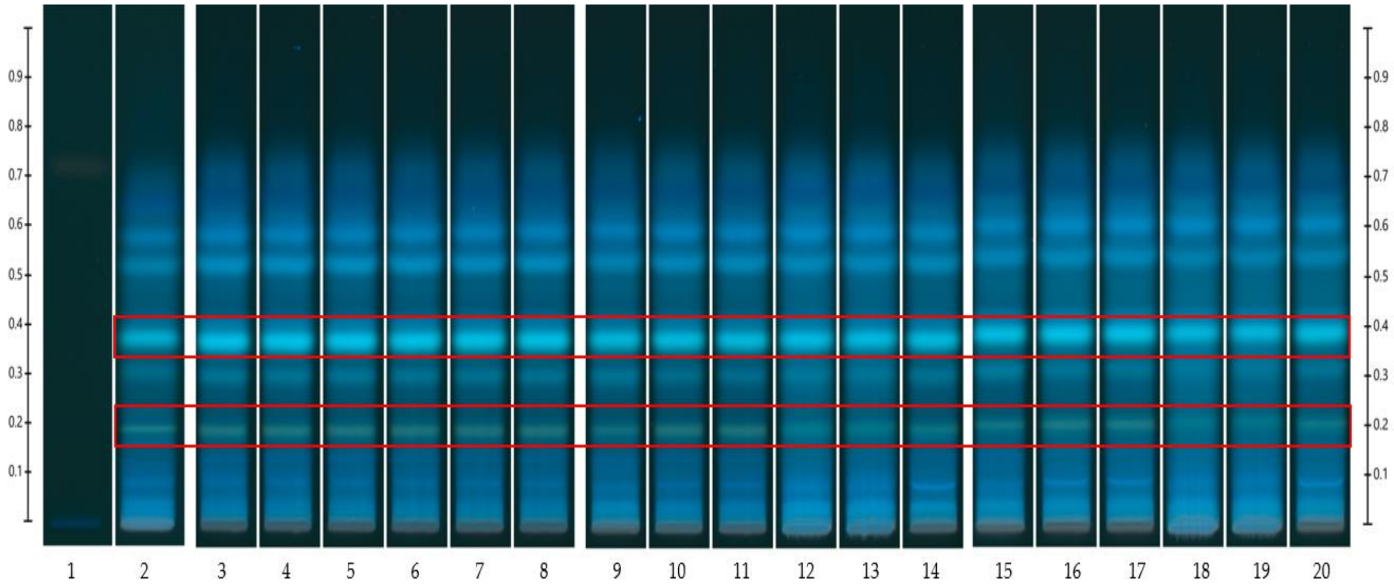

Figure S8. WAM2 honey - Red box indicates the monitored bands at Rf 0.20 and 0.38; Track 1 - 4,5,7-trihydroxyflavone (internal standard), Track 2- WAM 2 honey extract (system suitability test), Tracks 3-8 - WAM2 dry sheet extract collected at 1-6 months storage at 5°C, Tracks 9-14 – WAM2 dry sheet extract collected at 1-6 months storage at 30°C, Tracks 15-20 – WAM2 dry sheet extract collected at 1-6 months storage at 40°C; Image taken at 366 nm.

Table S10. Peak area of selected bands of Jarrah honey and WA Manuka honey 2 and their respective formulations (n=3, data represents mean ± SD).

Honey	Storage temperature (°C)	Sample	Weight (g)	Rf of Monitored Compound	Peak area (AU x 10^{-3}) per band					
					1 month	2 month	3 month	4 month	5 month	6 month
		Neat honey	1.01	0.20	5.3±0.01	5.3±0.02	5.3±0.02	5.3±0.02	5.2±0.01	5.3±0.01
				0.53	15.2±0.02	15.2±0.02	15.2±0.02	15.2±0.02	15.2±0.02	15.2±0.02
	5	Pre-gel	1.02	0.20	5.3±0.03	5.3±0.03	5.3±0.04	5.3±0.04	5.2±0.03	5.3±0.03
				0.53	15.2±0.02	15.2±0.02	15.2±0.03	15.2±0.02	15.2±0.03	15.2±0.02
WA Jarrah		Wet sheet	1.01	0.20	5.2±0.01	5.2±0.01	5.2±0.01	5.2±0.01	5.2±0.01	5.2±0.01
				0.53	14.8±0.03	14.8±0.03	14.8±0.03	14.8±0.03	14.8±0.03	14.8±0.03
		Dry sheet	1.02	0.20	14.0±0.04	14.0±0.02	14.0±0.02	14.0±0.02	14.0±0.02	14.0±0.02
				0.53	40.8±0.03	40.8±0.03	40.7±0.03	40.8±0.03	40.7±0.03	40.8±0.02
		Neat honey	1.02	0.20	5.2±0.02	5.3±0.02	5.2±0.02	5.3±0.02	5.2±0.01	5.2±0.03
				0.53	15.2±0.02	15.2±0.03	15.2±0.02	15.2±0.02	15.2±0.03	15.2±0.02
		Pre-gel	1.01	0.20	5.2±0.02	5.2±0.03	5.2±0.03	5.2±0.02	5.2±0.03	5.2±0.02
	30			0.53	15.2±0.02	15.2±0.02	15.2±0.03	15.2±0.02	15.2±0.03	15.2±0.02
		Wet sheet	1.02	0.20	5.1±0.01	5.1±0.01	5.1±0.02	5.1±0.01	5.1±0.02	5.1±0.02
				0.53	14.8±0.03	14.8±0.03	14.8±0.02	14.8±0.03	14.8±0.02	14.8±0.03
		Dry sheet	1.02	0.20	14.1±0.03	14.1±0.02	14.1±0.02	14.1±0.02	14.1±0.02	14.1±0.03
				0.53	40.8±0.03	40.8±0.03	40.7±0.03	40.8±0.03	40.7±0.03	40.8±0.02
		Neat honey	1.01	0.20	5.2±0.01	5.2±0.02	5.2±0.02	5.2±0.02	5.2±0.01	5.2±0.01
				0.53	15.2±0.01	15.2±0.02	15.2±0.01	15.2±0.02	15.2±0.02	15.2±0.01
		Pre-gel	1.02	0.20	5.2±0.03	5.2±0.03	5.2±0.04	5.2±0.02	5.2±0.03	5.2±0.02
	40			0.53	15.2±0.02	15.2±0.02	15.2±0.03	15.2±0.02	15.2±0.03	15.2±0.02
		Wet sheet	1.01	0.20	5.1±0.02	5.1±0.01	5.1±0.01	5.1±0.02	5.1±0.01	5.1±0.02
				0.53	14.8±0.02	14.8±0.02	14.8±0.03	14.8±0.03	14.8±0.02	14.8±0.03
		Dry sheet	1.01	0.20	14.0±0.03	14.1±0.02	14.0±0.03	14.0±0.02	14.0±0.02	14.0±0.02
				0.53	40.8±0.02	40.8±0.03	40.8±0.03	40.8±0.03	40.8±0.03	40.8±0.02
		Neat honey	1.01	0.20	17.4±0.03	17.4±0.03	17.4±0.03	17.4±0.04	17.4±0.03	17.4±0.03
				0.38	23.5±0.03	23.5±0.04	23.5±0.03	23.5±0.04	23.5±0.04	23.5±0.02
		Pre-gel	1.02	0.20	17.3±0.03	17.3±0.03	17.3±0.02	17.3±0.03	17.3±0.02	17.3±0.03
WA Manuka 2				0.38	23.4±0.02	23.4±0.02	23.4±0.01	23.4±0.02	23.4±0.01	23.4±0.03
	5	Wet sheet	1.02	0.20	17.1±0.03	17.1±0.03	17.1±0.04	17.1±0.04	17.1±0.03	17.1±0.04
				0.38	23.3±0.02	23.3±0.04	23.3±0.04	23.3±0.03	23.3±0.04	23.3±0.03
		Dry sheet	1.01	0.20	47.5±0.03	47.5±0.04	47.5±0.03	47.5±0.04	47.5±0.02	47.5±0.03
				0.38	64.9±0.02	64.9±0.03	64.9±0.02	64.9±0.03	64.9±0.02	64.9±0.02

		Neat honey	1.02	0.20	17.4±0.03	17.4±0.03	17.4±0.03	17.4±0.04	17.4±0.03	17.4±0.03
				0.38	23.5±0.03	23.5±0.04	23.5±0.03	23.5±0.04	23.5±0.04	23.5±0.02
		Pre-gel	1.01	0.20	17.3±0.03	17.3±0.03	17.3±0.02	17.3±0.03	17.3±0.02	17.3±0.03
				0.38	23.4±0.02	23.4±0.02	23.4±0.01	23.4±0.02	23.4±0.01	23.4±0.02
	30	Wet sheet	1.02	0.20	17.2±0.03	17.2±0.03	17.2±0.04	17.2±0.04	17.2±0.03	17.2±0.04
				0.38	23.3±0.02	23.3±0.04	23.3±0.04	23.3±0.03	23.3±0.04	23.3±0.03
		Dry sheet	1.02	0.20	47.6±0.03	47.6±0.04	47.6±0.03	47.6±0.04	47.6±0.02	47.6±0.03
				0.38	65.0±0.02	65.0±0.03	65.0±0.02	65.0±0.03	65.0±0.02	65.0±0.03
		Neat honey	1.01	0.20	17.4±0.03	17.4±0.03	17.4±0.03	17.4±0.04	17.4±0.03	17.4±0.03
				0.38	23.5±0.02	23.5±0.04	23.5±0.03	23.5±0.04	23.5±0.04	23.5±0.02
		Pre-gel	1.02	0.20	17.3±0.03	17.3±0.03	17.3±0.02	17.3±0.03	17.3±0.02	17.3±0.03
	40			0.38	23.4±0.03	23.4±0.02	23.4±0.01	23.4±0.02	23.4±0.01	23.4±0.03
		Wet sheet	1.02	0.20	17.1±0.03	17.1±0.03	17.1±0.04	17.1±0.02	17.1±0.03	17.1±0.02
				0.38	23.3±0.02	23.3±0.04	23.3±0.04	23.3±0.03	23.3±0.04	23.3±0.03
		Dry sheet	1.02	0.20	47.5±0.03	47.5±0.03	47.5±0.03	47.5±0.03	47.5±0.02	47.5±0.03
				0.38	64.9±0.03	64.9±0.03	64.9±0.02	64.9±0.03	64.9±0.02	64.9±0.02

Table S11. Peak area of selected bands in wet and dry sheets of Jarrah and WA Manuka honey (n=3, data represents mean ± SD).

Honey	Storage temperature (°C)	Sample	Weight (g)	Rf of Monitored Compound	Peak area (AU x 10^{-3}) per sheet					
					1 month	2 month	3 month	4 month	5 month	6 month
		Wet sheet	1.01	0.20	130±0.02	130±0.02	130±0.01	130±0.02	130±0.02	130±0.01
WA Jarrah	5			0.53	370±0.02	370±0.02	370±0.03	370±0.02	370±0.03	370±0.02
		Dry sheet	1.02	0.20	126±0.01	126±0.02	126±0.01	126±0.02	126±0.02	126±0.01
				0.53	367±0.03	367±0.03	366±0.02	367±0.02	366±0.03	367±0.02
		Wet sheet	1.02	0.20	128±0.02	128±0.02	128±0.02	127±0.02	128±0.02	128±0.02
	30			0.53	370±0.03	371±0.03	370±0.03	370±0.03	371±0.03	370±0.03
		Dry sheet	1.01	0.20	127±0.03	127±0.03	127±0.03	127±0.03	127±0.03	127±0.02
				0.53	367±0.02	367±0.03	368±0.02	367±0.02	368±0.02	367±0.02
		Wet sheet	1.02	0.20	128±0.02	127±0.02	128±0.02	127±0.02	128±0.02	127±0.02
	40			0.53	369±0.02	369±0.02	369±0.02	369±0.02	369±0.02	369±0.02
		Dry sheet	1.01	0.20	126±0.03	127±0.03	126±0.03	127±0.03	126±0.03	127±0.01
				0.53	367±0.02	368±0.02	368±0.02	367±0.01	367±0.02	367±0.02
		Wet sheet	1.01	0.20	427±0.02	427±0.02	426±0.03	427±0.02	426±0.03	427±0.02
WA	5			0.38	582±0.03	582±0.03	583±0.03	582±0.03	582±0.03	583±0.03
Manuka 2		Dry sheet	1.01	0.20	428±0.03	428±0.03	427±0.02	428±0.03	427±0.02	428±0.03
				0.38	584±0.03	585±0.03	584±0.03	585±0.03	584±0.02	584±0.03
		Wet sheet	1.02	0.20	428±0.02	428±0.02	428±0.02	428±0.02	428±0.02	428±0.02
	30			0.38	581±0.03	582±0.03	581±0.03	582±0.03	581±0.02	581±0.03
		Dry sheet	1.02	0.20	427±0.03	428±0.03	427±0.03	428±0.03	427±0.03	427±0.03
				0.38	585±0.02	585±0.02	584±0.02	585±0.02	584±0.02	585±0.02
		Wet sheet	1.01	0.20	427±0.03	428±0.03	427±0.03	427±0.03	428±0.03	427±0.03
	40			0.38	582±0.02	581±0.02	581±0.02	582±0.02	581±0.02	582±0.02
		Dry sheet	1.02	0.20	427±0.02	426±0.02	426±0.02	427±0.02	426±0.02	427±0.02
				0.38	584±0.02	584±0.02	585±0.02	584±0.02	585±0.02	584±0.02

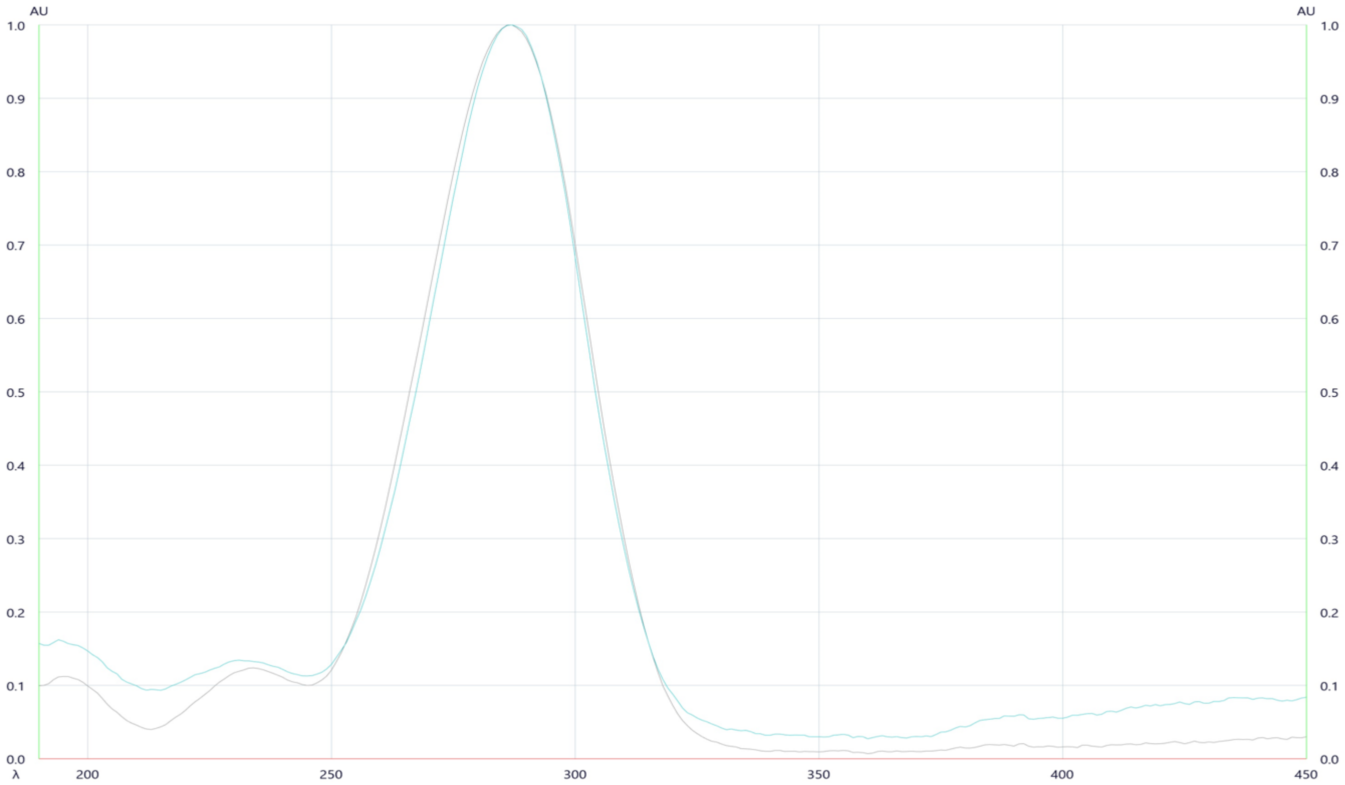

Figure S9. Absorbance spectra of HMF aqueous solution (grey line) and honey extract (blue line).
